# Sovateltide (IRL-1620) activates neuronal differentiation and prevents mitochondrial dysfunction in adult mammalian brains following stroke

**DOI:** 10.1038/s41598-020-69673-w

**Published:** 2020-07-29

**Authors:** Amaresh K. Ranjan, Seema Briyal, Anil Gulati

**Affiliations:** 1 Chicago College of Pharmacy, Midwestern University, Downers Grove, IL 60515 USA; 2 Pharmazz Inc. Research and Development, Willlowbrook, IL USA

**Keywords:** Neuroscience, Diseases of the nervous system, Stroke, Stem cells, Adult stem cells, Neural stem cells, Cerebrovascular disorders, Stroke

## Abstract

The development of effective drugs for stroke is urgently required as it is the 2nd largest killer in the world and its incidence is likely to increase in the future. We have demonstrated cerebral endothelin B receptors (ETBR) as a potential target to treat acute cerebral ischemic stroke. However, the mechanism of ETBR mediated neural regeneration and repair remains elusive. In this study, a permanent middle cerebral artery occluded (MCAO) rat model was used. Sovateltide (an ETBR agonist) injected intravenously showed better survival and neurological and motor function improvement than control. Higher neuronal progenitor cells (NPCs) differentiation along with better mitochondrial morphology and biogenesis in the brain of sovateltide rats were noted. Exposure of cultured NPCs to hypoxia and sovateltide also showed higher NPC differentiation and maturation. This study shows a novel role of ETBR in NPCs and mitochondrial fate determination in cerebral ischemia, and in improving neurological deficit after stroke.

## Introduction

Endothelin B receptors (ETBR) are G-protein coupled receptors and are expressed abundantly in vascular and neural cells and are also known to be involved in the central nervous system (CNS) development, neural cell survival and proliferation^1–3^. Their role in developing CNS, neuronal proliferation and migration as well as in the expression of angiogenic growth factors have been demonstrated with ontological studies^4,5^. Their deficiency at the prenatal stage of life resulted in fatal birth defects in the animal models, while their deficiency at the post-natal stage resulted in an increase in apoptosis and decrease in NPC within the dentate gyrus and cerebellum of the brain^2,4,6^. ETBR play a crucial role not only in the early developmental stages of the CNS, but its stimulation following CNS damage later in life has also been shown to enhance neurogenesis and angiogenesis, thereby promoting CNS repair^7–9^. We demonstrated that selective stimulation of ETBR by its agonist, IRL-1620 (INN approved by WHO is sovateltide), could significantly improve neurological and motor functions, with concurrent decrease in infarct volume and oxidative stress damage following permanent middle cerebral artery occlusion (MCAO) in rats^10,11^. We have also shown that administration of sovateltide in newborn rat pups increased vascular endothelial growth factor (VEGF) but had no effect on nerve growth factor (NGF) in postnatal developing brains^5^. Moreover, sovateltide treatment after cerebral ischemia protected neurons by enhancing angiogenesis, as we noted an increase in neuronal nuclei number and VEGF expression in the ischemic brain tissues. Additionally, animals receiving sovateltide displayed increased number of proliferating cells as well as NGF^+^ cells in the infarcted brain tissues^12^. Recently, we confirmed sovateltide as a pro-survival and anti-apoptotic agent in ischemic brains of MCAO rats^13^. To assess its safety and efficacy in human subjects, we performed a phase I clinical trial (CTRI/2016/11/007509) in healthy human volunteers^14^ and a multicentric, randomized, double-blind, parallel, saline-controlled efficacy clinical trial (NCT04046484) in patients with acute cerebral ischemic stroke^15^. Another multicentric, randomized, blinded, controlled efficacy clinical trial (NCT04047563) in 110 acute ischemic stroke patients is ongoing.

We have consistently shown in our preclinical studies carried out using MCAO rat model^10–13^, that sovateltide treatment helps in restoration of blood flow, modulation of apoptosis and improvement in neurological and motor functions after cerebral ischemic stroke. These observations indicate a role of ETBR in tissue healing after acute ischemic damage in the brain after stroke. However, the mechanism of healing after stimulation of ETBR by sovateltide in stroked brain remains elusive. In mammalian organs, tissue healing is mainly mediated through the process of regeneration, which is a complex process and involves multiple pathways including inflammatory, apoptotic, fibrotic, angiogenic, hypoxic and most importantly tissue plasticity. The tissue plasticity is mediated by the differentiation potential of the stem/progenitor cells, which can be readily activated after injury to produce mature cells. In brain, the differentiation of neural progenitor (NP) cells is known to be regulated mainly post-transcriptionally^16^. Neuronal ELAV (embryonic lethal, abnormal vision) family proteins, HuB, HuC and HuD are known to regulate the stability of RNAs of cell cycle suppressors as well as neuronal differentiation and maturation markers in NP cells^17^. Consequently, they can stop the mitotic phase and start differentiation in NP cells using the post-transcriptionally regulated switches. Hu family proteins are known to play an important role during prenatal and postnatal brain development and has been shown to play essential roles in learning and memory. Moreover, HuD is known to regulate the expression of master regulator of neuronal differentiation, NeuroD1^18^. Hence, expression analysis of HuC/HuD and NeuroD1 can be used as a tool for assessment of differentiation potential of NPCs in the brain after stroke. Moreover, Doublecortin (DCX) is known to be expressed in various stages of differentiation of NPCs and serves as an indicator of NPCs population in brain^19^.

Also, the complete regeneration of an organ includes its functional restoration after damage and mitochondria plays an important role in the neuronal cell survival as well as in its function^20^. Hence, assessment of mitochondrial fate (fusion, fission and biogenesis) and morphology is important for determining the functional status of neural cells in the brain after stroke.

To examine whether sovateltide can lead to neuronal regeneration and repair in brain after acute ischemia, we investigated the differentiation potential of NPCs and mitochondrial fate in the brain after stroke. We used the MCAO rat model and treated them with intravenous injections of sovateltide and examined the expression of DCX in brain tissues to assess the effect of stroke on neuronal cell population in the brain at 24 h post MCAO. Expression of differentiation markers of neuronal progenitor cells (NPCs), HuC/HuD and Neuro D1was also examined in these tissues. NeuroD1 is known to regulate the late stage of neuronal differentiation and expression of a mature neuronal marker, NeuN. To evaluate the neuronal differentiation potential of sovateltide, in hypoxic (ischemic) condition, we exposed cultured adult rat neural progenitor cells to hypoxia and sovateltide, and examined the expression of NeuroD1 and NeuN after 24 h. To determine the mitochondrial fate in ischemic brain tissues we assessed mitochondrial fusion and fission with western blotting and immunofluorescence at 24 h and day 7 post MCAO. Expression of the fission marker, DRP1 and fusion marker, MFN2 was evaluated. Effect of fusion and fission of mitochondria in brain tissues was further assessed by their morphological analysis with transmission electron microscopy (TEM). We assessed the mitochondrial biogenesis in the stroked brain using in situ PCR technique to detect mitochondrial DNA content in brain tissues.

## Results

### Sovateltide treatment improves survival as well as neurological and motor function after ischemic stroke

**Figure 1:**
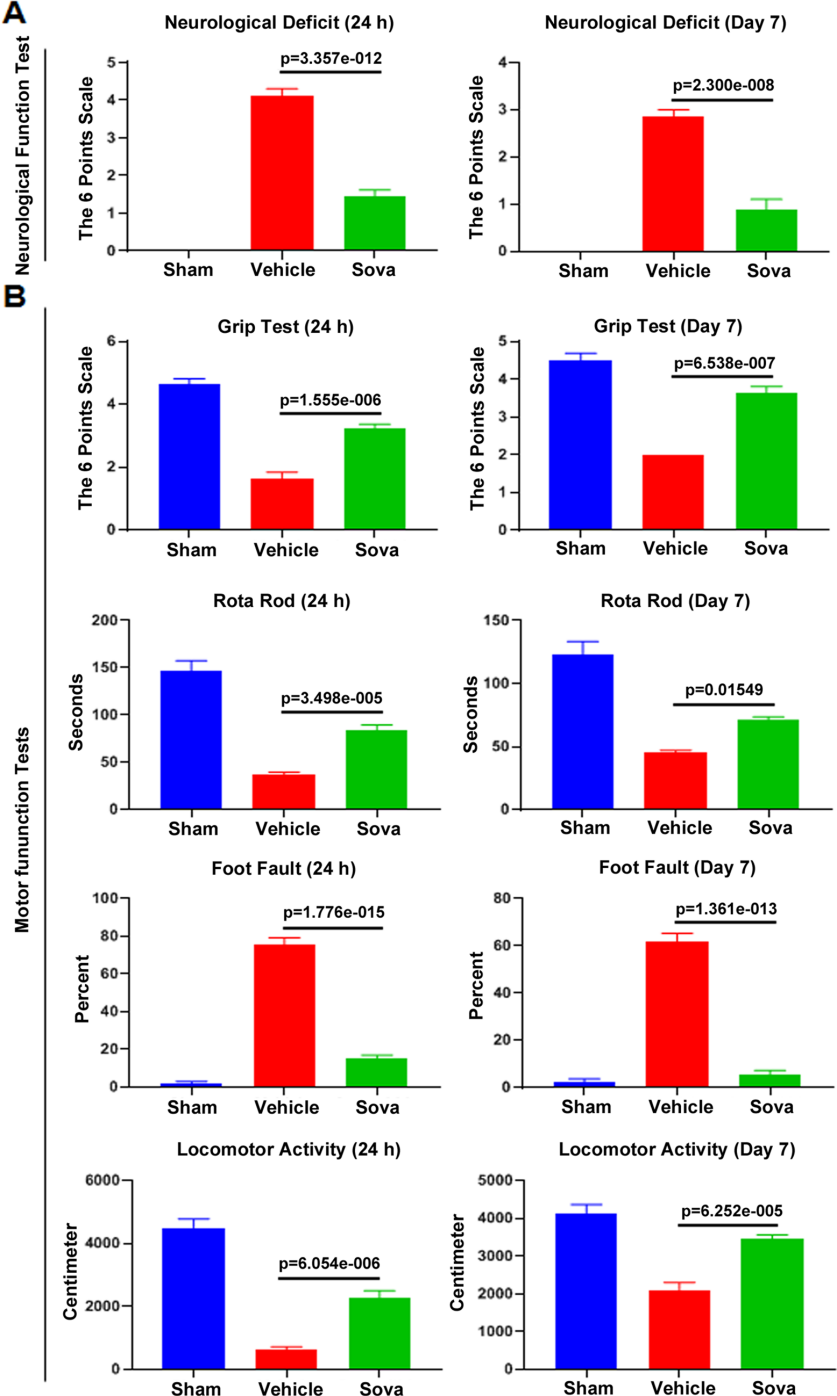
Effect of sovateltide on neurological and motor performance in the MCAO rats. Data with Mean ± SEM were plotted (supporting data tables are provided in Table S1).

We observed mortality in MCAO + vehicle rats at 24 h post MCAO, however no mortality in MCAO + sovateltide group at 24 h post MCAO was observed. The survival rate of 77.33% (11/15) in MCAO + vehicle, while 100% (9/9) survival rate in MCAO + sovateltide group was observed at 24 h post MCAO (Table 1). Increased 7-day survival was observed in MCAO + sovateltide than MCAO + vehicle group. Survival on day 7 post MCAO was 43.75% (7/16) in vehicle and 88.89% (8/9) in sovateltide treated rats. No mortality was observed in sham treated rats at either time points (Table 1). Sovateltide treatment after acute cerebral ischemic stroke improved the rate of survival. The neurological and motor function testing in rats were carried out before MCAO, and at 24 h and day 7 post MCAO (Table S1). The neurological deficit was scored at the 6-point scale (0 indicating no deficit and 5 indicating death). Significantly lower neurological deficit in sovateltide compared to vehicle treated rats was observed at 24 h (1.44 ± 0.18 vs. 4.07 ± 0.18, mean diff. − 2.618, 95% CI − 3.088 to − 2.148, p < 0.0001) and day 7 post MCAO (0.88 ± 0.23 vs. 2.86 ± 0.09, mean diff. − 1.982, 95% CI of diff. − 2.449 to − 1.516, p < 0.0001) (Fig. 1A). A significant improvement in all the tested motor functions was also observed in sovateltide compared to vehicle treated MCAO rats (Fig. 1B). Grip test score improved (3.22 ± 0.15 vs. 1.64 ± 0.17, mean diff. 1.586, 95% CI of diff. 1.063 to 2.109, p < 0.0001) at 24 h post MCAO and (3.63 ± 0.18 vs. 2.00 ± 0.0, mean diff. 1.625, 95% CI of diff. 1.150 to 2.100, p < 0.0001) at day 7 post MCAO in sovateltide vs vehicle. Rota-rod test (83.56 ± 6.04 vs. 36.60 ± 2.12, mean diff. 46.96, 95% CI of diff. 27.83 to 66.08, p < 0.0001) at 24 h post MCAO and (71.38 ± 2.09 vs. 45.57 ± 0.94, mean diff. 25.8, 95% CI of diff. 5.463 to 46.14, p < 0.015) at day 7 post MCAO was significantly improved in sovateltide compared to vehicle. An improvement in foot fault (15.21 ± 1.63 vs. 75.78 ± 2.86, mean diff. − 60.57, 95% CI of diff. − 67.75 to − 53.39, p < 0.0001) at 24 h post MCAO and (5.49 ± 1.68 vs. 61.71 ± 2.36, mean diff. − 56.23, 95% CI of diff. − 62.93 to − 49.52, p < 0.0001) at day 7 post MCAO was seen with sovateltide compared to vehicle. Locomotor activity (2,245 ± 253 vs. 601 ± 78, mean diff. 1623, 95% CI of diff. 1,038 to 2,209, p < 0.0001) at 24 h post MCAO and (3,451 ± 106 vs.. 2,103 ± 131, mean diff. 1,347, 95% CI of diff. 789 to 1905, p < 0.0001) at day 7 post MCAO in sovateltide compared to vehicle was observed. These results indicate significant effect of sovateltide in improving neurological and motor functions following MCAO in rats.

**Table 1:** Survival of rats after MCAO and sovateltide treatment.

Survival					
Sham		MCAO + vehicle		MCAO + sovateltide	
24 h post MCAO	Day 7 post MCAO	24 h post MCAO	Day 7 post MCAO	24 h post MCAO	Day 7 post MCAO
8/8	8/8	11/15	7/16	9/9	8/9
100.00%	100.00%	77.33%	43.75%	100.00%	88.89%

### Sovateltide treatment rescues neuronal progenitor cell population in acute cerebral ischemic stroked brain

**Figure 2:**
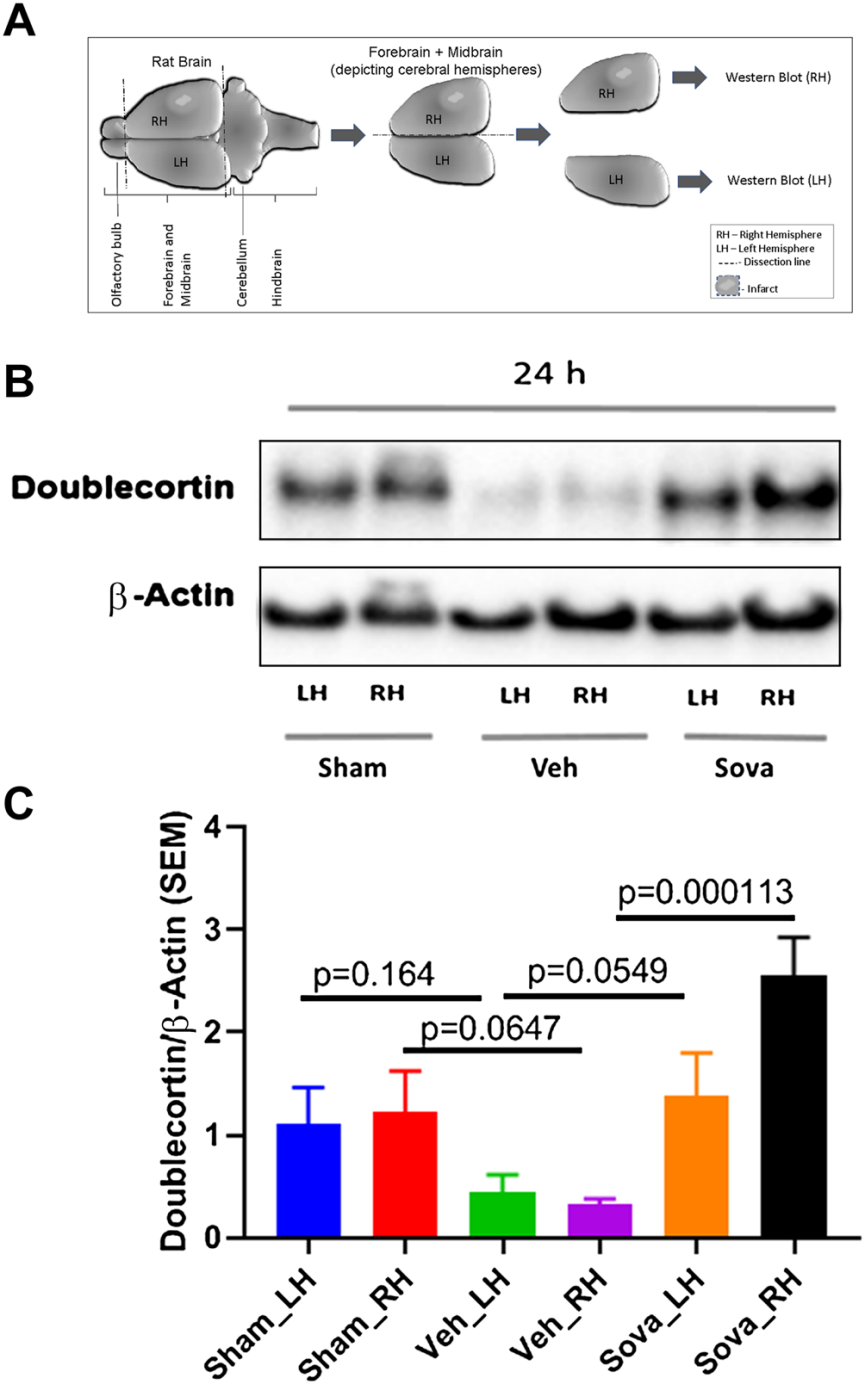
Expression of neural progenitor marker Doublecortin (DCX) in stroked brain and after sovateltide treatment. (**A**) Diagrammatic representation of adult rat brain isolation and tissue collection for western blots. (**B**,**C**) Blots and densitometry graphs of sham, vehicle and sovateltide (Sova) treated rat right hemisphere (RH) and left hemisphere (LH) brain tissues at 24 h post MCAO. All blots are representative of four different experiments with similar results in rat brains. Values are expressed as mean ± SEM. β-Actin was developed after re-probing of Doublecortin blots with anti-β-Actin and used as a loading control and normalization (full blots in Fig. S1A).

Doublecortin (DCX) represents a pan neuronal progenitor marker, which expresses in NPCs at different stages ranging from very early stages to the late stages of the neuronal differentiation. Hence, its level of expression in brain tissues would closely represent the corresponding amount of NPCs population. Since, the neurological and functional tests showed that stroke due to MCAO caused significant neurological and motor function deficit in rats compared to sham, while treatment with sovateltide rescued these deficits as soon as 24 h post MCAO. To assess whether the neurological deficit is correlated with the fate of NPCs in MCAO rat brains, we examined rat brain tissues of 24 h post MCAO using western blots. We observed significantly (p = 0.064) decreased level of DCX expression in the right (ischemic) cerebral hemisphere of MCAO + vehicle rats at 24 h post MCAO (Fig. 2B,C), which reflected the adverse effect of stroke on NPCs population. However, treatment of MCAO rats with sovateltide significantly (p = 0.0001) increased the expression of DCX (Fig. 2B,C and Fig. S1A) compared to vehicle treated rats. These results suggest that sovateltide treatment can rescue the NPCs population in damaged brain following acute ischemic stroke.

### Sovateltide promotes expression of post-transcriptional differentiation regulators HuC/HuD after acute ischemic stroke in MCAO rats

**Figure 3:**
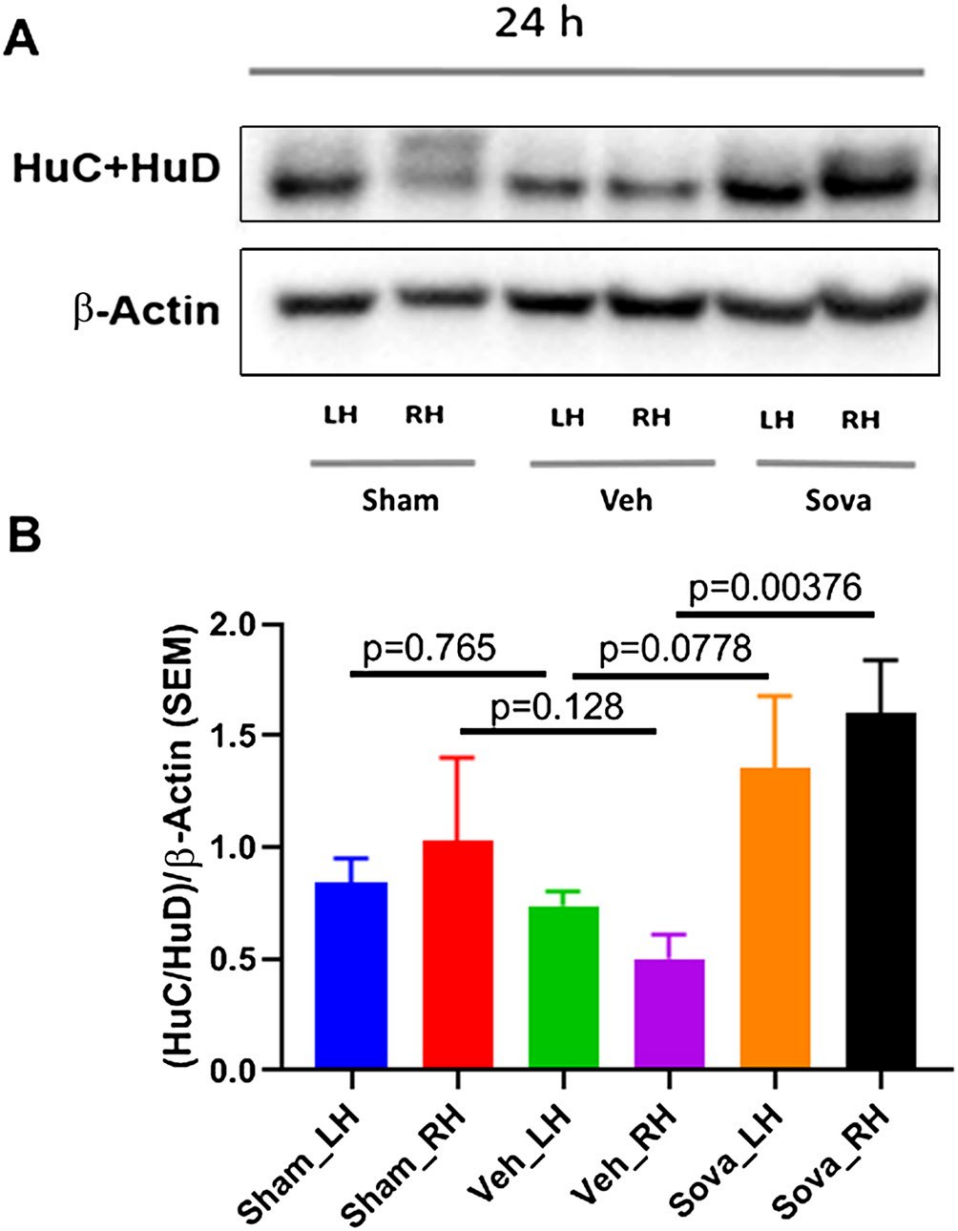
Expression of NPCs differentiation marker HuC/HuD in stroked brain and after sovateltide treatment. (**A**,**B**) western blots and densitometry graphs of sham, vehicle and sovateltide (Sova) treated rat right hemisphere (RH) and left hemisphere (LH) brain tissues at 24 h post MCAO. All blots are representative of four different experiments with similar results in rat brain. Values are expressed as mean ± SEM. β-Actin was developed after re-probing of HuC + HuD blots with anti-β-Actin and used as a loading control and normalization (full blots in Fig. S1B).

HuC/HuD are important RNA binding proteins and are known to regulate the differentiation and maturation of NPCs post-transcriptionally. We observed no change in the expression of HuC/HuD in the left (p = 0.765) or right (p = 0.128) cerebral hemispheres of “MCAO + vehicle” rats compared to sham (Fig. 3A,B) at 24 h post MCAO. While, sovateltide treatment significantly (p = 0.0037) increased the expression of HuC/HuD in the right hemisphere of MCAO rats at 24 h post MCAO. Also, a slight but insignificant increase (p = 0.077) in HuC/HuD in the left cerebral hemisphere of MCAO + sovateltide rats compared to that of MCAO + vehicle rats was observed. (Fig. 3A,B and Fig. S1B). These results indicate that sovateltide mediated ETBR activation could be involved in post-transcriptional regulation of NPCs differentiation and maturation in acute ischemic brain.

### Sovateltide induces expression of NeuroD1, a master regulator of neuronal differentiation

**Figure 4:**
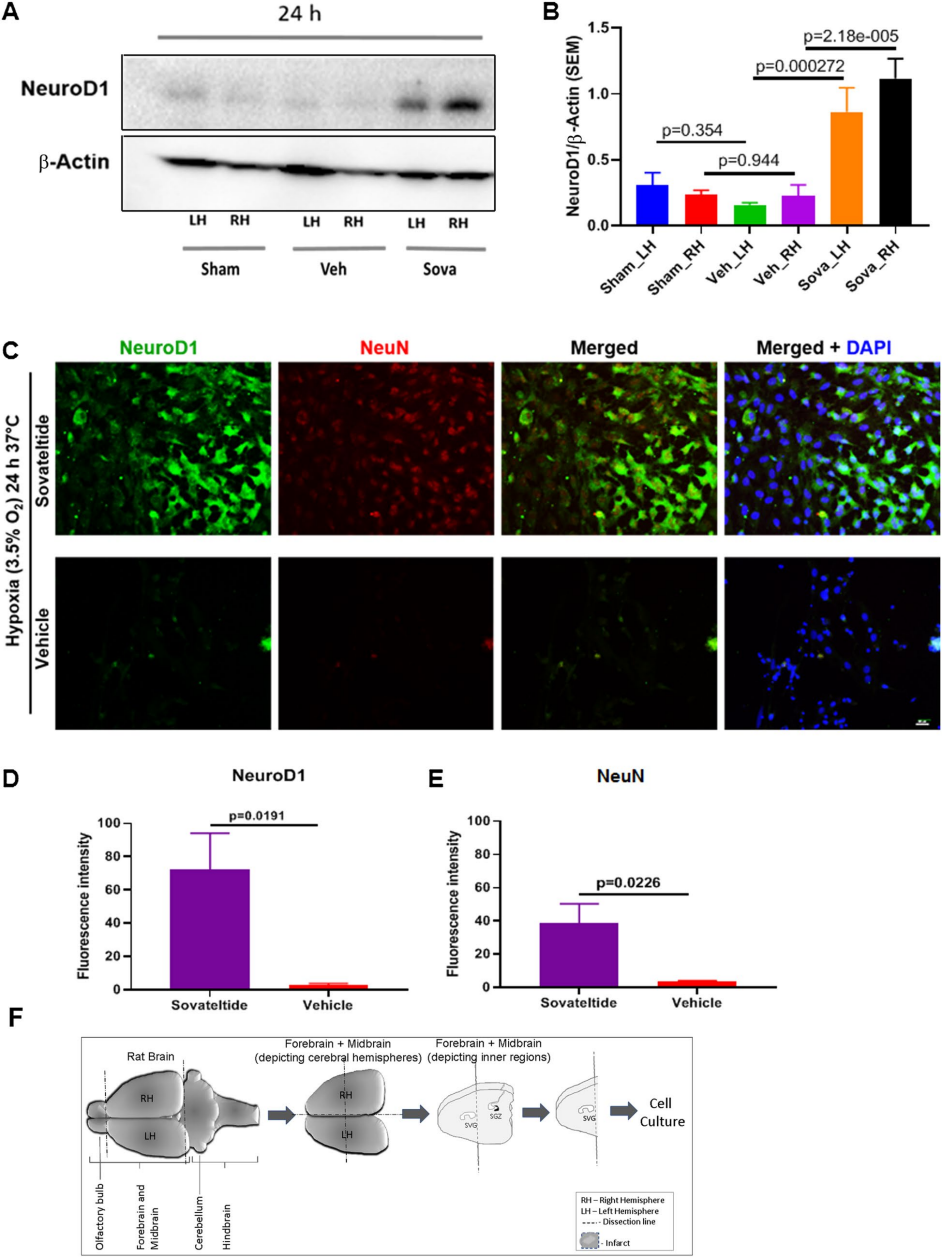
Expression of NPCs differentiation marker NeuroD1 in stroked brain and after sovateltide treatment. (**A**,**B**) western blots and densitometry graphs of sham, vehicle and sovateltide (Sova) treated rat right hemisphere (RH) and left hemisphere (LH) brain tissues at 24 h post MCAO. All blots are representative of four different experiments with similar results in rat brain. Values are expressed as mean ± SEM. β-Actin was developed after re-probing of NeuroD1 blots with anti-β -Actin and used as a loading control and normalization (full blots in Fig. S1C). (**C**) Expression of neuronal differentiation marker, NeuroD1 in cultured adult rat neural progenitor cells in hypoxia. Representative immunofluorescence microscopy images of sovateltide (1 ng/ml) treated cultured neural progenitor cells after 24 h of hypoxia exposure (supporting images are provided in Fig. S2). Higher expression of NeuroD1 (green) and mature neuronal marker, NeuN (red) was observed in sovateltide than vehicle treated samples. Nuclei were stained with DAPI (blue). Bar scale = 75 µm. (**D**,**E**) Fluorescence intensity graphs of NeuroD1 (**D**) and NeuN (**E**). Values are expressed as mean ± SEM. (**F**) Diagrammatic representation of dissection of adult rat brain and tissue collection for cell culture.

HuD protein is known to have a positive feedback loop relationship with a transcription factor and AT-rich DNA binding protein, SATB1, which directly influence the expression of NeuroD1. We assessed the expression of NeuroD1 using western blots and observed very low level of expression in brains of sham as well as “MCAO + vehicle”, however sovateltide treated MCAO rats showed significantly up-regulated expression of NeuroD1 in right (p = 0.000022) as well as left (p = 0.00027) cerebral hemisphere (Fig. 4A,B and Fig. S1C), which also suggested a role of sovateltide mediated ETBR signaling in promoting neuronal differentiation in acute ischemic brain. To further examine the expression of NeuroD1 and differentiation of NPCs into mature neurons, cultured NP cells were treated with sovateltide and exposed to hypoxia for 24 h at 37 °C. A higher expression of NeuroD1 (green, Fig. 4C,D) in sovateltide treated NPCs than vehicle treated NP cells was observed. Moreover, higher expression of NeuN, a mature neuronal marker (red, Fig. 4C,E), in sovateltide treated cells was observed compared to vehicle (Fig. S2). These results suggest an early regenerative response of sovateltide with accelerating the differentiation of NP cells into mature neuronal cells after cerebral ischemic stroke.

### Sovateltide regulates mitochondrial fission/fusion in stroked brain

**Figure 5:**
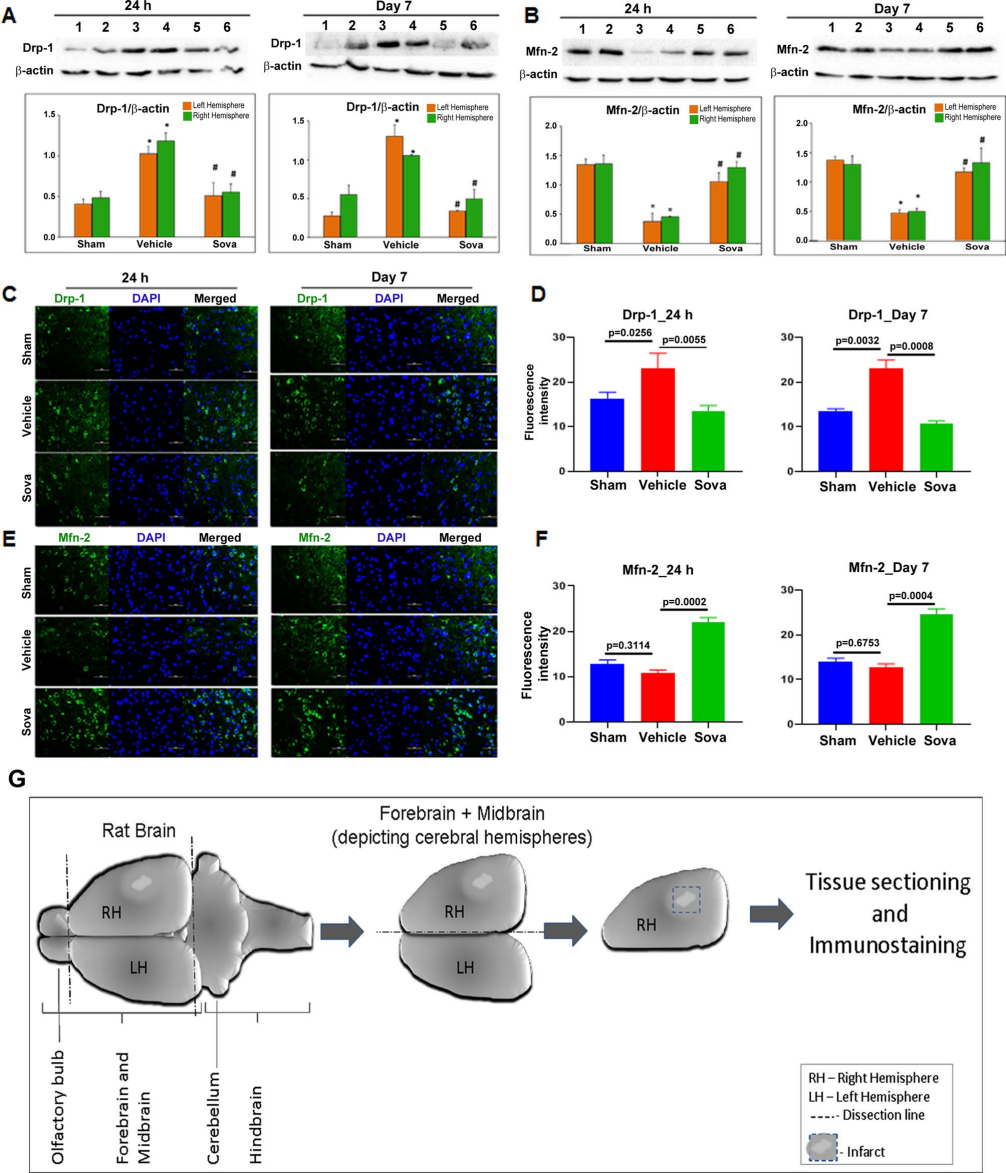
Expression of mitochondrial fission and fusion markers in stroked brain tissues. (**A**,**B**) western blots and densitometry graphs, (**C**,**D**) immunofluorescence images of fission marker (Drp1) and fusion marker (Mfn 2) in sham, vehicle and sovateltide (Sova) treated rat right hemisphere (RH) and left hemisphere (LH) brain tissues at 24 h and day 7 post MCAO. (**A**,**B**) densitometry values are expressed as mean ± SEM and β-Actin was developed after re-probing of protein blots with anti-β-Actin and used as a loading control and normalization (full blots in Fig. S3). *p < 0.001 compared to sham, ^#^p < 0.0001 compared vehicle. (**C**,**E**) representative immunofluorescence images showing expression of Drp 1 (green, **C**) and Mfn 2 (green, **E**). Nuclei were stained with DAPI (blue), Scale bar = 20 µm (**C**,**E**). n = 4. (**D**,**F**) fluorescence intensity graphs of Drp 1 (**D**) and Mfn 2 (**F**) at 24 h and day 7 post MCAO. Values are expressed as mean ± SEM. (**G**) Diagrammatic representation of dissection of adult rat brain and tissue collection for tissue sectioning and immunostaining.

**Figure 6:**
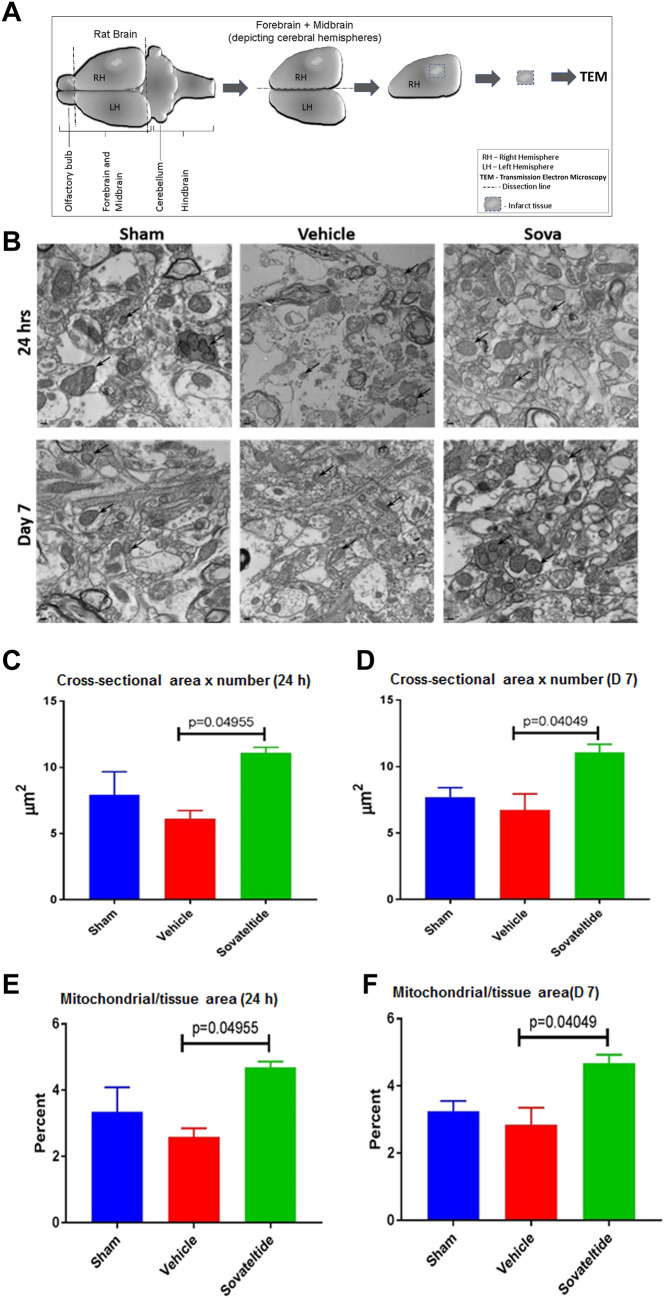
Transmission electron microscopic (TEM) analysis of mitochondria in brain tissues. (**A**) Diagrammatic representation of dissection of adult rat brain and tissue sectioning for TEM. (**B**) representative TEM images of sham, vehicle and sovateltide (Sova) treated rat right hemisphere (RH) brain tissues at 24 h and day 7 post MCAO (supporting images are provided in Fig. S4). Black arrows indicate representative mitochondria. Image magnification 3,000×, scale bar = 0.2 µm. (**B**) Measurement of mitochondrial cross-sectional area × number at 24 h and day 7 post MCAO, respectively. (**C**) Measurement of percent mitochondrial to tissue area ratio at 24 h and day 7 post MCAO, respectively. Values are expressed as mean ± SEM (**B**,**C**).

Western blot analyses showed a significantly higher expression of mitochondrial fission marker, Drp1 (p < 0.001) and reduced expression of mitochondrial fusion marker, Mfn2 (p < 0.001) in vehicle treated MCAO rat brains compared to sham at 24 h and day 7 post MCAO (Fig. 5A). On the other hand, a significantly reduced expression of fission marker Drp1 (p < 0.0001) and increased expression of the fusion marker Mfn2 (p < 0.0001) was observed in sovateltide than vehicle treated rat brain tissues at 24 h and day 7 post MCAO (Fig. 5B and Fig. S3). The qualitative immunofluorescence data of these markers in the right (ischemic) cerebral hemisphere also showed lower expression of Drp1 (green, Fig. 5C,D) and higher expression of Mfn2 (green, Fig. 5E,F) in sovateltide than vehicle treated samples. These results indicated that ischemic stroke caused mitochondrial fission or dysfunction in brain tissues, which was alleviated by sovateltide. To further validate changes in mitochondrial fission and fusion, we examined brain tissues with transmission electron microscopy and observed significant increase in mitochondrial “cross-sectional area × number” (p < 0.05) and “percent mitochondrial/tissue area” (p < 0.05) in sovateltide treated rat brains at 24 h as well as day 7 post MCAO (Fig. 6B–F and Fig. S4).

### Sovateltide treatment promotes mitochondrial biogenesis in ischemic brain after stroke

**Figure 7:**
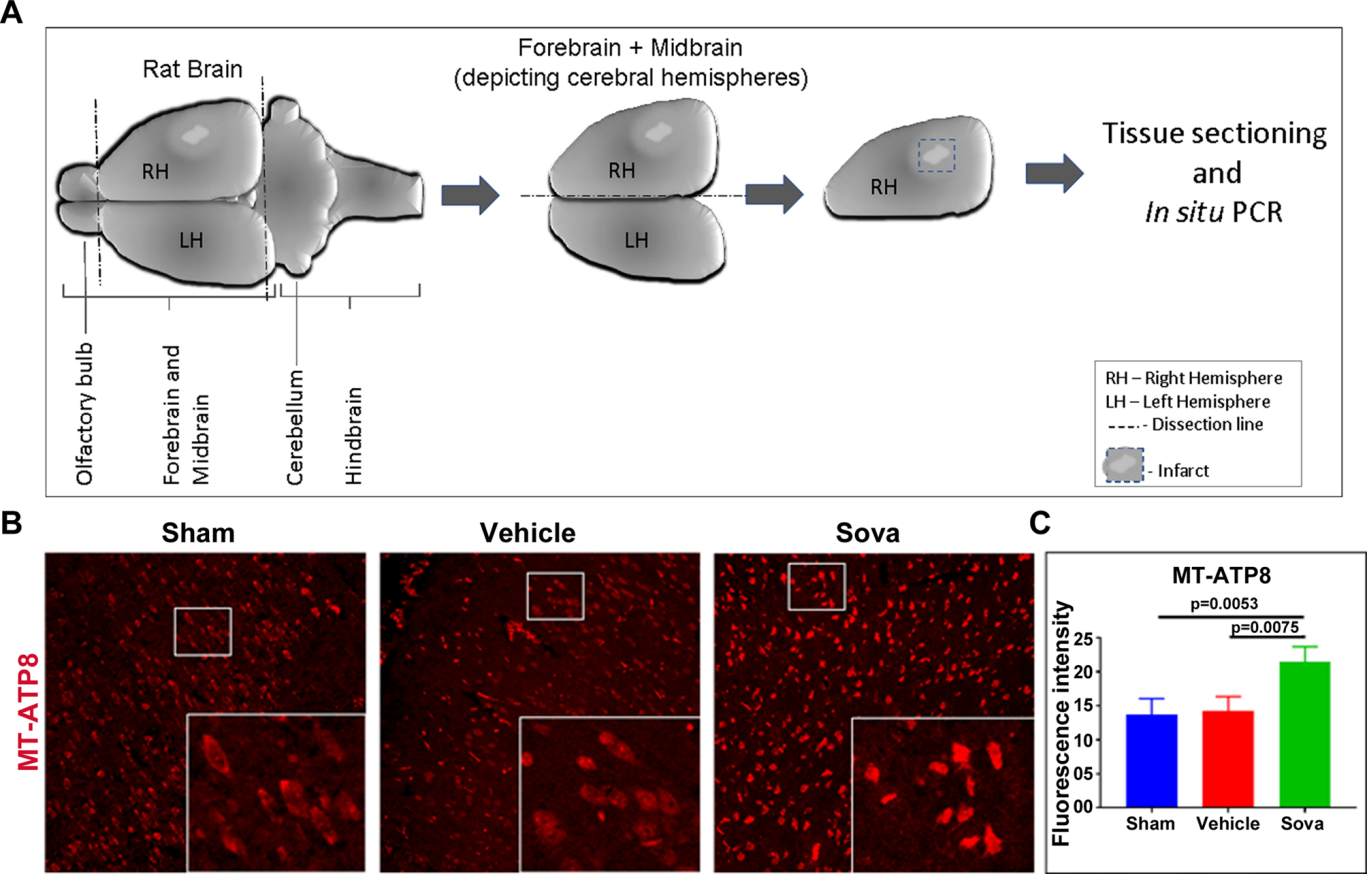
In situ PCR analysis of mitochondrial biogenesis in brain tissues. (**A**) Diagrammatic representation of dissection of adult rat brain and tissue sectioning for in situ PCR. (**B**) representative in situ PCR images of sham, vehicle and sovateltide (Sova) treated rat right hemisphere brain tissues at day 7 post MCAO (supporting images are provided in Fig S5). Red fluorescence indicates amplified MT-ATP8 DNA in mitochondria. Image magnification 200×. (**B**) fluorescence intensity graph of MT-ATP8 DNA. Values are expressed as mean ± SEM.

Mitochondrial biogenesis is known to play important roles in survival and function of neural cells. To assess mitochondrial biogenesis after sovateltide treatment in ischemic brains we carried out in situ PCR on fixed brain tissues at day 7 post MCAO to detect mitochondrial DNA. We used Taqman probe/primers for MT-ATP8 gene, which is present exclusively in mitochondrial genome. The probe was VIC labeled and the amplified PCR product was detected as VIC fluorescence using a confocal microscope. We observed significantly higher MT-ATP DNA fluorescence (red) in sovateltide treated brain tissues than vehicle (p = 0.0075) treated samples (Fig. 7B,C, Fig. S5). These results indicated higher copy number of mitochondrial genome, suggesting their improved biogenesis in sovateltide treated brain tissues than vehicle.

## Discussion

Acute cerebral ischemic stroke causes damage to brain tissues, which may lead to disabilities or death. According to recent statistics, stroke kills an average of 140,000 Americans annually^21^. Tissue plasminogen activator (tPA) and stentriever to remove thrombi are primary treatment options with limited success. Therefore, efforts to develop better therapies are continuously being made. However, due to complex pathophysiology of stroke, development of a new promising drug is challenging as evident by failure of several agents in late stage clinical trials^22^. The cerebral ischemic pathophysiology involves hypoxia, vascular damage, inflammation, apoptosis and other events, which may cause neural cell damage and functional impairment of the brain. Therefore, investigating a target protein or receptor, which regulates multiple cellular functions in neural cells, would be useful for discovering effective drugs to treat stroke. We have studied ETBR in the brain, which are known to regulate numerous functions including blood perfusion, cell proliferation, cell survival, apoptosis, differentiation, neurogenesis and neural development^1–3,9,23–25^. We used sovateltide, a highly selective agonist of ETBR to treat acute cerebral ischemic stroke and observed its beneficial effects in our pre-clinical studies in MCAO rats and clinical efficacy trial (NCT04046484) of acute cerebral ischemic stroke patients.

In the current study, we used a well-established MCAO rat model of stroke in our laboratory. We permanently occluded the middle cerebral artery to create focal ischemic stroke in the right cerebral hemisphere of the rat brain. This procedure of MCAO interrupts the blood flow mainly in the right hemisphere of the brain; however, the partial ischemic effects on the contralateral cerebral hemisphere due to interference in collateral blood flow is possible^13^. We treated one set of MCAO rats at 4, 6, and 8 h and sacrificed them at 24 h post MCAO, while another set of rats were treated similarly at day 0, 3 and 6 and sacrificed at day 7. Rats were subjected to neurological and motor function evaluation and we observed improved neurological and motor functions in sovateltide than vehicle treated rats at 24 h and day 7 post MCAO. An improvement in these neurological and motor functions might be because of prevention of further damage and repair of brain tissue after sovateltide treatment of MCAO rats. Since, ischemic stroke causes damage to brain tissues and therefore their regeneration is prerequisite for the functional restoration of the brain after stroke^26^. Hence, we were interested in exploring whether sovateltide helped in regeneration of brain tissues after acute cerebral ischemic stroke. The brain tissue regeneration involves angiogenesis as well as generation of new neural cells^27,28^. Several studies have shown presence of NP cells in the adult brain and demonstrated as a potential neural cell source^29–33^. The NP cells are known to be stimulated after CNS damage and differentiated into neural cells^34–36^. The differentiation and migration of NP cells/NPCs are essential for generation of new mature neuronal cells and replacement of scarred/fibrotic tissues. These responses in early stage of tissue repair is required to avoid the fibrotic events, which otherwise may lead to replacement of damaged neuronal cells with fibrotic cells resulting in scarring/fibrosis and compromised neural function in the affected area.

We analyzed rat brain tissues at 24 h post MCAO using western blotting technique and assessed the expression DCX to determine the effect of MCAO induced acute ischemic stroke on NPCs population. DCX expression has been shown in various stages of neuronal differentiation of NPCs, ranging from NPCs at very early stage (granular cells) to the late stage of differentiation in the brain. DCX is mainly known to be expressed in the highly committed neuronal progenitors with very low multipotency and thus its expression level could represent the overall status of NPCs in brain after stroke and treatment. However, a very small percentage of DCX^+(low)^ NPCs have been shown with ability to differentiate into cells other than neurons. To assess their differentiation to neuronal cells we examined the neuronal differentiation markers HuC/HuD and NeuroD1. HuC and HuD proteins belong to the ELAV (embryonic lethal, abnormal vision)/Hu protein family. They are RNA binding proteins and are known to interact with AU rich instability sequences / elements (ARE) present on the 3′ UTR of target RNAs. Binding of Hu proteins on the targeted RNAs provides stability to RNAs and helps in their translation to express respective proteins. Their targets include several RNAs responsible for neuronal differentiation, maturation, migration, growth, survival and cell cycle regulators (e.g. SATB1, MARCKS, GAP 43, Tau, AChE, NOVA 1, NGF, BDNF, NT-3 and P21^cip^^1^^/waf1^). The knock down studies of HuC have shown its role in learning and memory e.g. neurological function. While, role of HuD have been demonstrated for neuronal differentiation, neurite growth and neuronal synapses^37–39^. The HuD knock out mice showed increased neurosphere (neural stem/progenitor) formation and decreased neuronal differentiation^40^. Recent study by Wang et al. has shown existence of a positive feedback loop between HuD and SATB 1^18^. HuD binds to the ARE of SATB 1 mRNA and increases its stability, while SATB 1 binds to the AT rich region of HuD gene and increases its transcription. Moreover, SATB 1 is also known to increase the expression of NeuroD1, a master regulator of neuronal differentiation.

**Figure 8:**
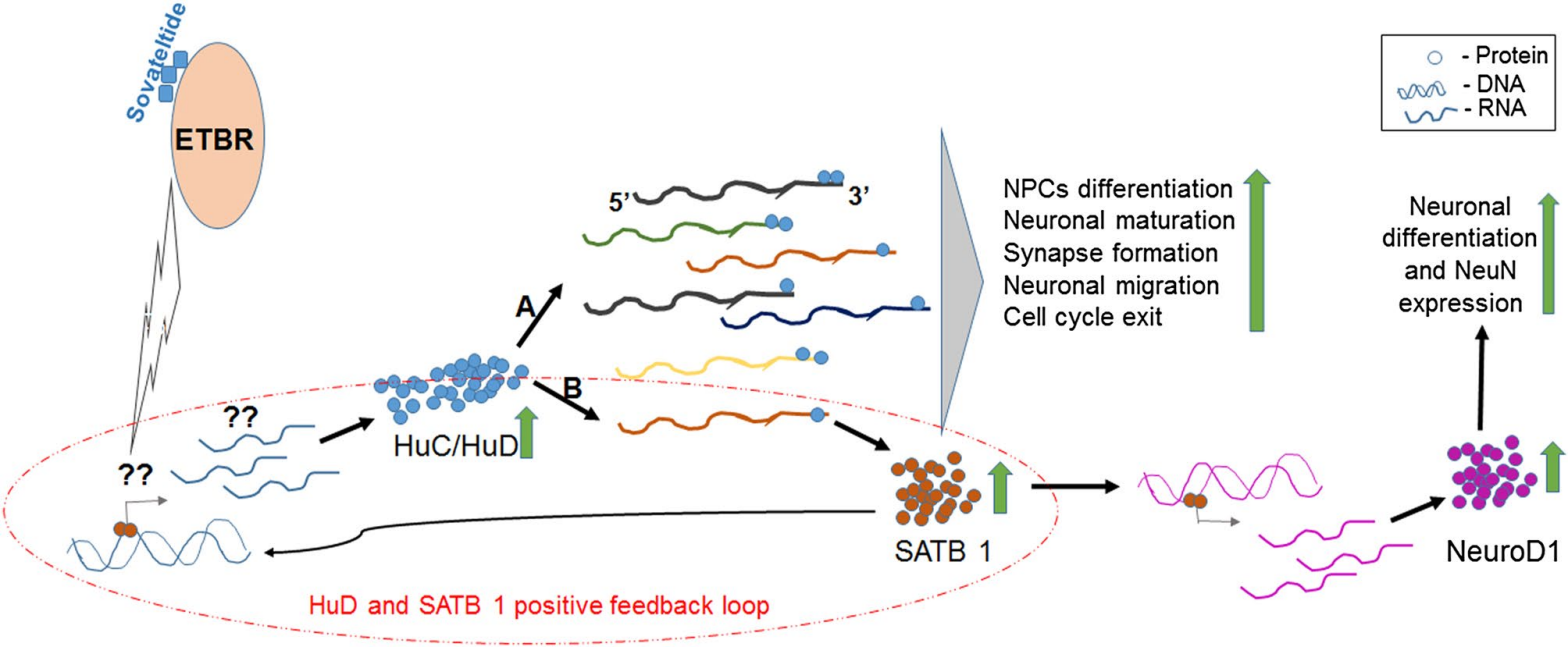
Plausible mechanism of action of ETBR signaling involved in neuronal differentiation of NPCs. Activation of ETBR signaling after sovateltide treatment increases HuC/HuD expression in NPCs probably (indicated with ‘??’ in the diagram) through increased transcription or stability of HuC/HuD RNAs. Increased expression of HuC/HuD would be pushing the NPCs towards neuronal differentiation with increasing the stability of RNAs involved mainly in NPCs differentiation, neuronal maturation and cell cycle exit (**A**). SATB 1 RNA is one of them, which is known to be stabilized after binding of HuD on its 3′ UTR. SATB 1 is a transcription factor and positively regulates the expression of NeuroD1, a master regulator of neuronal differentiation and also of HuD (positive feedback loop, shown in broken red circle) (**B**).

We examined expression of neuronal progenitor marker -DCX, and differentiation markers, HuC/HuD and NeuroD1 in MCAO rat brains. We observed significantly decreased expression of DCX and HuC/HuD in MCAO + vehicle rats, which suggested decreased NPCs population as well as their differentiation potential after stroke. However, sovateltide treatment induced expression of DCX, HuC/HuD and NeuroD1 in the right ischemic cerebral hemispheres of rat brains at 24 h post MCAO, which suggests a role of sovateltide in maintenance and differentiation of NPCs to generate new neural cells after acute ischemia (Fig. 8). The expression of NeuroD1 is known as a critical event in the neuronal differentiation and it has been shown that ectopic expression of NeuroD1 in epithelial or glial cells is sufficient to differentiate them into neuronal like cells^41,42^. It has also been demonstrated that NeuroD1 regulates the expression of a neuronal maturation marker, NeuN in NPCs^43^. To examine whether sovateltide helps in differentiation of NPCs we exposed cultured adult rat NP cells to hypoxia (3.5% oxygen) and 1 ng/ml sovateltide for 24 h. We observed significantly higher expression of NeuroD1 and NeuN compared to vehicle treated NP cells. We have shown the role of ETBR on neuronal cell proliferation in vivo by using the ETBR agonist, sovateltide (IRL-1620) with or without an ETBR antagonist, BQ 788 in our previous study^12^. We observed 45–50% increase in BrdU and NeuN positive cells in the infarcted cerebral hemisphere of sovateltide (IRL-1620) treated MCAO rats compared to vehicle treated rats. While, pretreatment with the ETBR antagonist, BQ788 blocked the effects of IRL-1620 on proliferation of neuronal cells. Thus, these observations support the role of sovateltide mediated ETBR signaling stimulation on differentiation of NPCs in ischemic/hypoxic condition. However, further studies are required to explore how sovateltide mediated ETBR signaling activation leads to increased expression of HuC/HuD and increases neuronal differentiation in NPCs after stroke.

Another aspect of our study was to explore the role of mitochondria in survival of NPCs and neuronal function restoration in stroked brain after sovateltide treatment. Our recent study has demonstrated that sovateltide treatment could modulate the interaction of apoptotic protein Bax to mitochondrial membrane^13^^,^ which is a critical event in mitochondria mediated cell death^44,45^. These observations suggested that sovateltide mediated activation of ETBR influenced the mitochondrial fate. Mitochondria are well known to regulate several important cellular processes including electron transfer and ATP generation, which are important for cell survival and function^46^. Therefore, healthy mitochondria and their function are some of the important determinants for cell survival, growth and regeneration. To study mitochondrial dysfunction, mitochondrial fission and fusion markers have been used in a number of studies and are like gold standard for initial assessment of mitochondrial fate^47^. Mitochondrial fission is evidently associated with dysfunction while fusion correlates with healthy and functional mitochondria. We assessed brain tissues and evaluated the level of fission and fusion markers by western blotting. We observed significantly lower level of fission marker (Drp1) and higher amount of fusion marker (Mfn2) in sovateltide than vehicle treated animals. Since, fission leads to fragmentation in mitochondria, which results in smaller sized mitochondria in cells while on the contrary the fusion results in increase in size of mitochondria. To measure the mitochondrial size in the tissue samples we carried out transmission electron microscopy (TEM). Mitochondria were identified as organelles with cristae in TEM images. We counted mitochondria and measured their diameter in sham, vehicle and sovateltide treated right cerebral hemisphere. The mitochondrial cross-sectional area was calculated using the formula лd^2^/4. Significantly increased values of “cross-sectional area × number” and “percent of mitochondrial area/tissue area” in sovateltide than vehicle treated right cerebral hemispheres were observed. Evaluation of mitochondrial “cross-sectional area × number” and “mitochondrial area/tissue area” helped us to understand the overall effective areas of mitochondria relative to the total brain tissue area examined in this study. The TEM data have helped us to closely examine the effect of mitochondrial fusion/fission in the tissue; however, it suffers from certain limitations such as difficulty in visualizing cristae in very small sized mitochondria resulted after fission. Moreover, it has been evidently shown that after cellular damage mitochondria become vacuolated^48–50^, which makes their identification even more difficult. In this study, we used n = 2 randomly selected samples from each treatment group for TEM, although the sample size for this experiment is small (n = 2) but the obtained results are quite convincing and have further supported the role of sovateltide in mitochondrial fusion (function) in neural cells in ischemic brain and warrants further investigations to understand the molecular mechanism and other associated phenomena in more detail. The mitochondrial biogenesis is one of such events, which is important for overall cellular function and is associated mainly with favorable and healthier cellular conditions. The biogenesis of mitochondria involves replication of mitochondrial DNA and increase in their copy number. To study biogenesis in fixed tissue samples we devised a technique of “in situ mitochondrial DNA PCR” by modifying our previously developed protocol of in situ PCR for detection of transcripts and miRNA^51^. We carried out in situ PCR using a mitochondrial gene; MT-ATP8 specific TaqMan probe and primers in day 7 post MCAO tissue samples. The MT-ATP8 gene specific TaqMan probe was labeled with VIC, which was detected by confocal microscope after PCR amplification and amount of amplified MT-ATP8 gene was estimated after analysis of acquired microscopic images. We selected MT-ATP8 gene because the MT-ATP8 gene is located exclusively in mitochondrial DNA. The mitochondrial genome is circular and contains only one copy of MT-ATP8 gene per circular genome in the mitochondria. Hence, essentially the amount of MT-ATP8 DNA fluorescence detected with in situ PCR represents the relative amount of mitochondrial circular DNA. We observed higher amount of mitochondrial DNA in sovateltide than vehicle treated right cerebral hemispheres. However, it was seen insignificantly different in vehicle than sham rats. Observation of similar fluorescence intensity of mitochondrial DNA in sham and vehicle is confounding and interesting as well. Moreover, similar results were observed through TEM (Fig. 6) in which “mitochondrial cross-sectional area × number” and “mitochondrial/tissue area” were insignificantly different in sham and vehicle. Thus, these observations suggest that although ischemic damage in the brain caused damage to neural tissues as well as mitochondrial fission (Fig. 5), mitochondria might not get eliminated from brain tissues immediately after ischemic damage of neural cells and would be floating around in damaged tissue for a while similar to existence of free mitochondria in heathy human blood sample^52^. The another possibility could be an influx of inflammatory cells in the damaged brain area, which might have compensated the loss of mitochondrial DNA signal due to neural cell damage in vehicle treated rats, while in sovateltide treated rats, mitochondria of inflammatory cells would be causing additional signal to that of mitochondria of protected neural cells. It would be an interesting subject to explore in our future studies.

Previous studies have demonstrated a role of endothelin signaling on mitochondrial function. Yuki et al., showed that mitochondrial dysfunction induced by rotenone, an inhibitor of mitochondrial respiratory chain complex I significantly increased the expression of ET-1 in cardiomyocytes, indicating that ET-1 may produce mitochondrial impairment in cardiomyocytes^53^. While, use of ET-A receptors antagonist, LU135252 has shown improvement in mitochondrial functions in failing canine hearts^54^. It has also been demonstrated that blockade of ETBR by BQ788 and A192621 induced mitochondrial dysfunction and intrinsic mitochondrial apoptotic pathway in glioma cell lines^55^, indicating roles of ETBR in mitochondrial function. The role of mitochondria in cerebral ischemia is also well known and has been described as a critical factor for neurological and motor function recovery after cerebral ischemic stroke^56,57^. However, the relationship between neural ETBR receptors and mitochondria in cerebral ischemia remains elusive^9^. To the best of our knowledge this is the first study to elucidate a potential link between the ETBR signaling and mitochondria in the brain after cerebral ischemic stroke.

Overall our current study has shown the role of sovateltide mediated activation of ETBR in neuronal regeneration with promoting the differentiation of neuronal progenitor cells and generation of mature neuronal cells after cerebral ischemic stroke. Moreover, it has also shown potential to protect the neural mitochondria and enhance their biogenesis, which may be critical events in protection and functional restoration of neural cells after cerebral ischemic stroke.

## Materials and methods

### Study design

#### Research subjects

Adult male Sprague–Dawley rats (Envigo, Indianapolis, IN) of weight range 350–390 g were used for this study.

#### Sample size (N)

The middle cerebral artery occlusion (MCAO) procedure causes stroke which may lead to death in MCAO rats. Our previous experience with MCAO indicated that it may cause death in ~ 50–60% rats within 7 days of MCAO, if remains untreated. In this study we included N = 8 in sham, N = 9 in MCAO + sovateltide and N = 15 in MCAO + vehicle for 24 h post MCAO, and N = 8 in sham, N = 9 in MCAO + sovateltide and N = 16 in MCAO + vehicle for day 7 post MCAO group at the start of the experiment. There was no mortality in rats at 24 h post MCAO, however there was mortality after 2nd day of MCAO and at day 7 post MCAO we were left with N = 8 in MCAO + sovateltide and N = 7 in MCAO + vehicle. No mortality in sham group at day 7 was observed.

#### Power analysis

Power analysis was conducted using the Statistics Calculator from A-priori Sample Size Calculator for Hierarchical Multiple Regression and data from stroke research confirmed that with a sample size of at least 6 in each group we would have a 80% power of detecting an expected change of 30% in all the marker levels with a significance level of 5% (two sided), taking Set A variables as the 3 time points and Set B variables as 1 degree of freedom to represent number of possible values of marker being detectable or not.

#### Randomization

After MCAO rats were randomized to receive either intravenous injection of sovateltide or normal saline.

#### Blinding

The neurological and motor function tests were performed and interpreted by investigators blinded to the treatment groups of rats.

#### Exclusion

Data of motor function test of day 7 post MCAO rats which died before day 7 were excluded for neurological as well as motor function tests. While motor function test data of rats died prior to 24 h post MCAO couldn’t be acquired.

#### Replication

Western blots were replicated four times while other techniques were replicated at least two times.

#### Guidelines

Results are reported according to the ARRIVE (Animal Research: Reporting of In vivo experiments) guideline listed in EQUATOR Network library.

#### Ethical aspects

All animal care and use for experimental procedures were performed in accordance with the guidelines of the Institutional Animal Care and Use Committee (IACUC) of Midwestern University. All biohazards were handled in accordance with Midwestern University Bio-safety Committee standard operating procedures and in compliance with OLAW/OSHA regulations.

### Animal experiment (MCAO and sovateltide treatment)

Male Sprague–Dawley rats (Envigo, Indianapolis, IN) of weight range 350–390 g were used for this study. Only male rats were used in this study to maintain consistency with previous studies and avoid female estrous cyclicity. Animal care, use of anesthetics and all the surgical procedure were approved by the IACUC of Midwestern University. The guidelines and regulations of the U.S. National Institute of Health were followed while performing all the animal procedure. Induction of focal cerebral ischemia via middle cerebral artery occlusion (MCAO) which was performed following the method described by Koizumi, et al.^58^ with modification of access route. In brief, rats were anesthetized with intraperitoneal (i.p.) injections of 100 mg/kg ketamine (Henry Schein Animal Health, Dublin, OH, USA) and 10 mg/kg of xylazine (Lloyd Laboratories, Shenandoah, IA, USA). A Cole Palmer Animal Monitoring Thermometer with colonic probe (Vernon Hills, IL, USA) was used to measure the rectal core temperature. The temperature at 37 ± 1 °C was maintained during surgery and recovery using the thermo-controlled base of the operating table. Animals were placed in a secure supine position and a midline incision was made to expose the right common carotid artery, external carotid artery, and internal carotid artery. Occlusion of middle cerebral artery was carried out by using a 4.0 monofilament nylon thread (CP Medical, Portland, OR, USA). The nylon thread was inserted from the external carotid artery and advanced into the lumen of the internal carotid artery until a resistance was felt (~ 20 mm). The nylon filament was left in place to create a permanent model of focal cerebral ischemia. In sham animals the common carotid artery and external carotid artery were exposed and the incision was sutured without touching the internal carotid artery^10,59^. After surgery animals were monitored continuously for recovery from anesthesia, and then observed twice per day until the end of the experiment. Rats were randomly divided into three groups (Group 1: Sham, Group 2: MCAO + Vehicle, Group 3: MCAO + Sovateltide). Sovateltide [N-Succinyl-[Glu9, Ala11,15] endothelin 1] (Pharmazz, Inc., Willowbrook, IL 60527, USA) was dissolved in saline and a dose of 5 µg/kg was administered (i.v.) into tail vein at 4, 6 and 8 h post MCAO. The dose of sovateltide was determined on the basis of its pharmacokinetics^60^ and our previous studies^10–12^.

### Neurological evaluation

Rats were subjected to a neurological evaluation prior to occlusion, and at 24 h, and day 7 post MCAO. We used a 6 point scale to assess neurological deficit as described in previous studies^11,59^. No neurological deficit is scored 0, while severe brain damage leading to death of rats is scored 5 at the 6 points scale.

### Motor performance tests

We used four different tests (1) grip test, (2) foot fault test, (3) rota-rod and (4) spontaneous locomotor activity test to assess the motor activity and coordination in MCAO rats. Rats were subject to these tests in a blinded manner prior to MCAO, and at 24 h and 7 days after MCAO.

#### Grip test

For the grip test a 50 cm long string was pulled taut between two vertical supports and was elevated 40 cm above a flat surface. A rat was placed on the midway of the string between the supports and evaluated for the grip according to a 6 point scale^11,59^.

#### Foot fault test

An elevated grid floor with a mesh size of 30 mm was used for the foot fault test. Rats were placed on the grid floor for one minute to acclimatize and then they were observed for 1 min and evaluated for foot fault errors as described earlier^11,59^.

#### Rota rod

Rats were placed on a rotating spindle of the rota rod apparatus (Rota-Rod 47700, Ugo Basile, Italy) and the time at which they fell off was recorded in seconds^11,59^.

#### Spontaneous locomotor activity

An animal activity meter (Opto-Varimex-4 Auto-Track System, Columbus Instruments, Columbus, OH) was used to test the spontaneous locomotor activity of the rats. Each rat was observed for 10 min in a square enclosed area equipped with infrared photocells along the X, Y, and Z axes to quantitatively measure spontaneous horizontal and vertical motion.

### Immunofluorescence

We used the immunofluorecence technique to detect expression of mitochondrial fission (DRP1) and fusion (MFN2) markers in rat brain tissues and to examine the expression of NeuroD1 and NeuN in cultured NPCs. The tissue immunofluorescence technique was followed as described earlier^7,13^. In brief, right brain hemisphere was dissected as depicted in Fig. 5G, and fixed in 50 ml of 4% paraformaldehyde (PFA) in NaPO_4_ buffer solution for 2 h at room temperature, and then submerged in 20% sucrose/4% PFA solution and stored at 4 °C for 48 h. Brain hemispheres were sliced into 10 µm thick slices using a cryostat (Microtome cryostat HM 505E; Walldorf, Germany) at − 20 °C. Tissue sections were washed three times with 1× PBS and permeabilized with 1% Triton-× 100 in PBS for 15 min at room temperature (RT). Blocking with 5% BSA in 1× PBS for 1 h at room temperature was carried out. The brain sections were incubated with anti-DRP1 and MFN2 antibody (1:200 diluted in 1× PBS) at 4 °C overnight. Sections were washed twice in 1× PBS and incubated with Alexa Fluor 488-conjugated donkey anti-rabbit secondary antibody (1:200, Abcam, Cambridge, MA) for 1 h at room temperature in the dark and mounted with prolong gold anti-fade reagent with DAPI (Cell Signaling Technology, Danvers, MA, USA).

The culture NPCs were fixed with 4% paraformaldehyde (PFA) in NaPO_4_ buffer solution for 30 min at room temperature. Washed twice with 1× PBS and permeabilized with 1% Triton- × 100 for 15 min at RT. Washed twice with 1× PBS and incubated with 4% BSA for 1 h at RT. After one wash with PBS cells were incubated with rabbit anti-NeuroD1 and mouse anti-NeuN antibodies after dilution of 1:200 in 1× PBS. Cells were incubated at 4 °C for overnight. Cells were washed 3 times with 1xPBS and incubated with Alexa Fluor 555-conjugated donkey anti-rabbit secondary antibody (1:400, Abcam, Cambridge, MA) for 1 h at room temperature in the dark and mounted with prolong gold anti-fade reagent with DAPI (Cell Signaling Technology, Danvers, MA, USA).

Fluorescence was detected using an inverted fluorescent microscope (Nikon Eclipse TiE, Melville, NY). All images for analysis were taken with the same exposure with a multi-channel ND acquisition using NIS Elements BR imaging software (Nikon Instruments, Inc., Melville, NY). Analyses was performed using NIS-Elements 3.01 imaging software from Nikon Instruments, Inc. (Melville, NY).

### Western blot

For western blot, whole rat brains were collected. Olfactory bulbs and hindbrain portions were dissected out and discarded. The right hemisphere (RH) and left hemisphere (LH) of the forebrain and midbrain were identified and dissected out as depicted in Fig. 2A. RH and LH were collected in different tubes and western blots were performed by using the lysate of RH and LH, separately. Western blot analysis on rat brain tissues was performed as described in our previous studies^10,11,13^. In brief, brain tissues were homogenized in RIPA buffer (20 mM Tris–HCl pH 7.5, 120 mM NaCl, 1.0% Triton X100, 0.1% SDS, 1% sodium deoxycholate, 10% glycerol, 1 mM EDTA and 1× protease inhibitor, Roche) after washing twice in chilled saline. Samples were centrifuged at 12,000 RPM at 4 °C for 30 min and supernatant was collected in new tubes. Protein concentration was determined using Folin-Ciocalteu’s Reagent. Protein (60 μg) was denatured in Laemmli sample buffer (Bio-Rad, Hercules, CA) with heating them in a boiling water bath for 5 min. Protein was resolved in 10% SDS-PAGE and transferred on nitrocellulose membranes (Sigma-Aldrich, St. Louis, MO, USA). The membranes were blocked with superblock or 4% BSA or 4% fat free milk solution for 1 h at room temperature. The membranes were incubated overnight with primary antibodies anti-Neuro D1, HuC/HuD, Doublecortin, DRP1 or MFN2, (1:1,000) (Abcam, Cambridge, MA, USA) at 4 °C overnight, followed by incubation with respective secondary antibodies goat anti-rabbit or goat anti mouse IgG, conjugated with horseradish peroxidase-(HRP) (1:2,000) (Santa Cruz Biotech., Santa Cruz, CA, USA) for 2 h at room temperature. β-actin (1:10,000; Sigma-Aldrich, St. Louis, MO, USA) was used as a loading control. The chemiluminescence of HRP was visualized with SuperSignal WestPico Chemiluminescent Substrate (Thermo Fisher Scientific, Bartlett, IL) using the Kodak Gel Logic 1500 Imaging System (CarestreamHealth Inc., New Haven, CT). The protein band intensity indicating the protein expression was analyzed using ImageJ (NIH) software and graphs were plotted after normalizing the protein expression with β-actin expression on their respective blots.

### Adult rat neural progenitor cell culture

The adult rats were anaesthetized, and brains were dissected out and collected in chilled saline. The dissected brains were processed immediately in sterile condition (inside the biosafety cabinet). A portion of cerebrum was dissected out as depicted in Fig. 4F, briefly cerebral hemisphere from the whole brain was isolated after dissecting out olfactory bubs as well as hind brain. The cerebral hemisphere was dissected again and front region of cerebral hemisphere including SVZ region. and the isolated portion of cerebrum was minced into small pieces with sterile scissors and centrifuged at 2,000 RPM at RT. The supernatant was discarded, and brain tissues were submerged in 0.2% trypsin EDTA solution and incubated at 37 °C for 20 min. The digested tissues were mixed properly with 1 ml pipette and filtered through the sieve of pore size of 70 µm. The filtered cells were mixed with 5 ml of serum containing neurobasal plus media (GIBCO) and centrifuged at 2,000 RPM at room temperature (RT) for 3 min. The supernatant was discarded, and cell pellet was washed twice with 1× PBS and with same settings of centrifugation. After washing, cell pellet was re-suspended in 10 ml Neurobasal Plus media with 10% FBS and 4× antibiotics (Penicillin and Streptomycin). Cells are counted with Neubauer’s counting chamber. Approximately 10^6^ cells per 100 mm tissue culture treated plate were seeded with 10 ml of media and incubated in the incubator with 5% CO_2_ and temperature 37 °C. After 24 h cells were collected and centrifuged at 2,000 rpm at RT for 3 min. Supernatant is discarded and cells are plated in new tissue culture treated plate with fresh media for 3 days. Adherent cells were observed after 3 days of culture, non-adherent cells were removed, and fresh media was added to the plate. The adherent neural progenitor cells were further cultured until plate gets confluent (6 to 8 days) with changing media every 3rd day.

### In situ PCR

In situ PCR was carried out to assess the mitochondrial biogenesis in the rat brain tissues, with following our previously developed in situ PCR protocol aimed to detect transcripts and miRNA in cultured cells^51^. In brief, the right brain hemisphere was dissected as depicted in Fig. 7A and was fixed in 4% paraformaldehyde. The fixed brain tissues were cryo-sectioned (10 µm thick) and sections were placed on 1% gelatin coated glass slides. Sterile molecular grade water (150–200 µl) was added to each tissue section and were incubated in − 80 °C for 5 min for freezing. The frozen section was quickly thawed at hot plate (40 °C) and the freeze thaw cycle was repeated 3 times. Blocking with 1% BSA made in sterile molecular grade water was done with incubation at RT for 20 min. BSA solution was removed and tissue sections were washed once with sterile molecular grade water. TaqMan Master mix (for 300 µl Master mix—75 µl of 2× cDNA reaction mix (Cat # QP9001, Alkali Scientific, Fort Lauderdale, FL) 15 µl of TaqMan MT-ATP8 probe/primer (Cat # 4448489, ThermoFisher Scientific, Grand Island, NY) and 210 µl of 20% glycerol in molecular grade water was prepared and 25 µl was added on each tissue section. Coverslip was carefully placed to avoid air bubble and it was sealed with a scotch tape from all sides to prevent evaporation of the reagent. The slides were placed over an aluminum foil with coverslip facing down and the aluminum foil was wrapped after placing a moist paper on top of the slide. The whole setting was placed in a thermal cycler with coverslip facing down and following cycle was set up. PCR cycle—Step 1. 95 °C for 5.5 min, cycle 1. Step 2. 95 °C for 30 s, 57 °C for 20 s, 72 °C for 40 s, cycle—39. Step 3. 72 °C for 2 min, cycle-1. Step 4. 7 °C for ∞. After completion of PCR (Step 3), the samples were washed with 1×PBS and coverslips were mounted again after adding antifade reagent. The tissue sections were imaged with confocal microscopy to detect the VIC fluorescence indicating amplified MT-ATP8 DNA in red channel. Images were acquired at 20× and fluorescence intensity was analyzed using the image J software.

### Transmission electron microscopy (TEM)

Rat brains were isolated after anesthetizing rats and a small portion (~ 1 cm^2^) of the right hemisphere of the brain covering the focal ischemic region in MCAO rat brain was quickly dissected out as depicted in Fig. 6A, and immersed in freshly prepared 2.5% glutaraldehyde + 2% paraformaldehyde tissue fixing solution in 1× PBS, pH 7.4. In case of sham, a similar sized portion of tissue was dissected out from the same position of the right hemisphere of brains and was fixed similarly. The tissue sample was incubated at RT for 30 min and then was stored in 4 °C until further processing for TEM as follows. The brain tissues were dissected into cubes of around 1 mm^3^ and processed with microwave assistance (Pelco Biowave, Ted Pella, Redding CA). Samples were rinsed twice in 0.1 M PIPES buffer, pH 7.4 followed by 0.1 M imidazole. Post fixation with 2% OsO_4_ in 0.1 M imidazole was followed by three rinses with deionized water (DI-H_2_O). Samples were dehydrated in an ascending series of 30%, 50%, 70%, 95% and 2 × 100% acetone. Infiltration with Embed812 resin: acetone dilutions of 3:1, 2:1, 1:1, 1:2, 3:1, and pure resin was carried out before transferring into fresh resin in silicon molds. Polymerization was done at 60 °C for 24 h. Ultrathin sections of 70 nm thickness were obtained with the Ultracut-S ultramicrotome (Leica, Buffalo Grove, IL) using a diamond knife (Diatome/EMS Hatfield, PA). Images were recorded with a 1,230 TEM (JEOL, Peabody, MA) at 100 kV acceleration voltage using an Orius camera (Gatan, Pleasanton, CA) at a magnification of 3,000×. Images were opened in the “Image J” software and mitochondria were counted and their diameter was measured with the help of line tool of “Image J” manually.

## Supplementary information

Supplementary Information 1.
