# Supplementary material for: Sovateltide (IRL-1620) activates neuronal differentiation and prevents mitochondrial dysfunction in adult mammalian brains following stroke
Source: Sci Rep. 2020 Jul 29;10:12737. doi: 10.1038/s41598-020-69673-w (PMC7391684; doi:10.1038/s41598-020-69673-w)

Title: Sovateltide (IRL-1620) activates neuronal differentiation and prevents mitochondrial dysfunction in adult mammalian brains following stroke

Short title – Sovateltide, neuronal differentiation and mitochondria

**Authors:** Amaresh K. Ranjan^1*^, Seema Briyal^1^, Anil Gulati^1,2*^

**Affiliations:**

1. Chicago College of Pharmacy, Midwestern University, Downers Grove, IL, 60515, USA
2. Pharmazz Inc. Research and Development, Willlowbrook, IL, USA

*Emails – [anil.gulati@pharmazz.com](mailto:anil.gulati@pharmazz.com), [aranja@midwestern.edu](mailto:aranja@midwestern.edu)

Supplementary Materials:

**Table S1. Neurological and motor function tests**

Neurological test

Motor function tests

**Figure S1. Full blot images of representative blots used in figures 2-4.** Full blot of Doublecortin and B-Actin (A), Full blot of HuC/HuD and B-Actin (B), and Full blot of NeuroD1 and B-Actin (C).


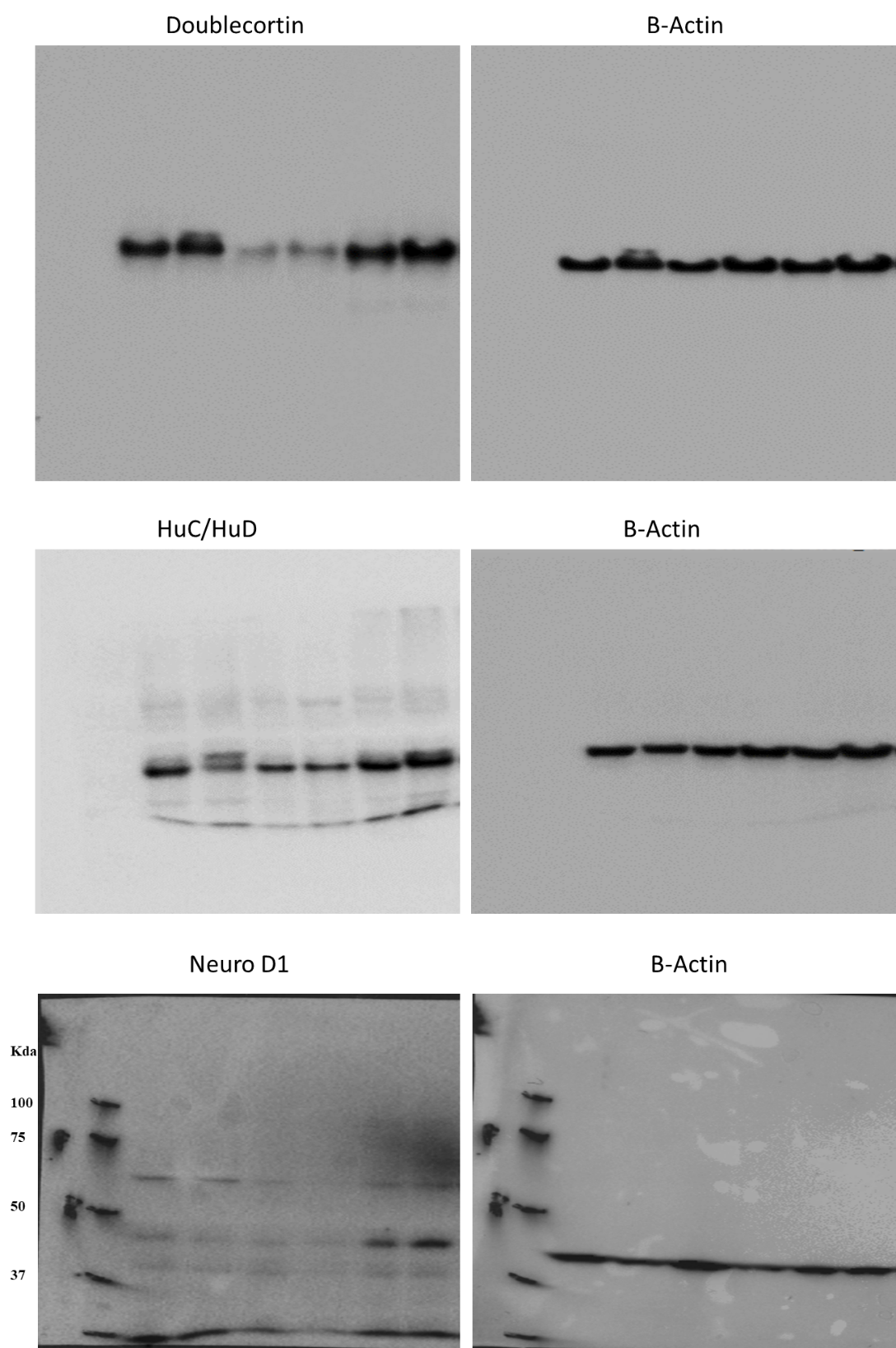


**A**

**B**

**C**

**Figure S2. Microscopy photographs of representative images used in figure 4C.** Multiple microscopy photographs of NPCs imaged from randomly selected wells of culture plate exposed to hypoxia in presence of sovateltide (A), and in presence of vehicle (normal saline) (B). NeuroD1 (green), NeuN (red) DAPI or Nuclei (blue) in cultured NPCs.

**A**

**B**


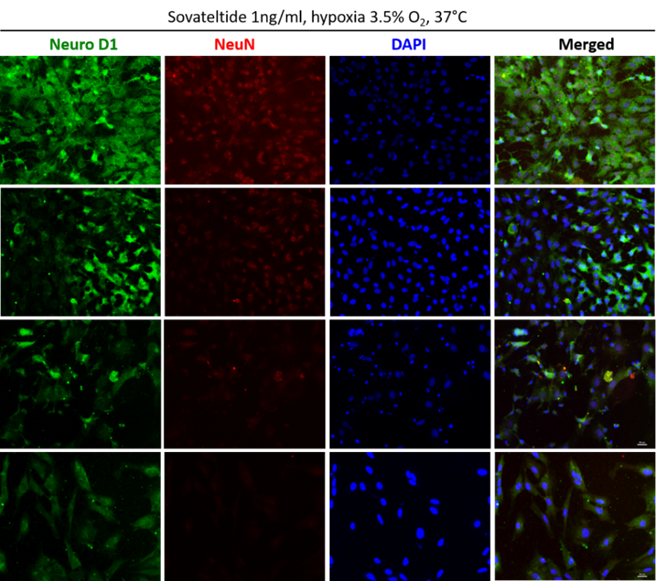


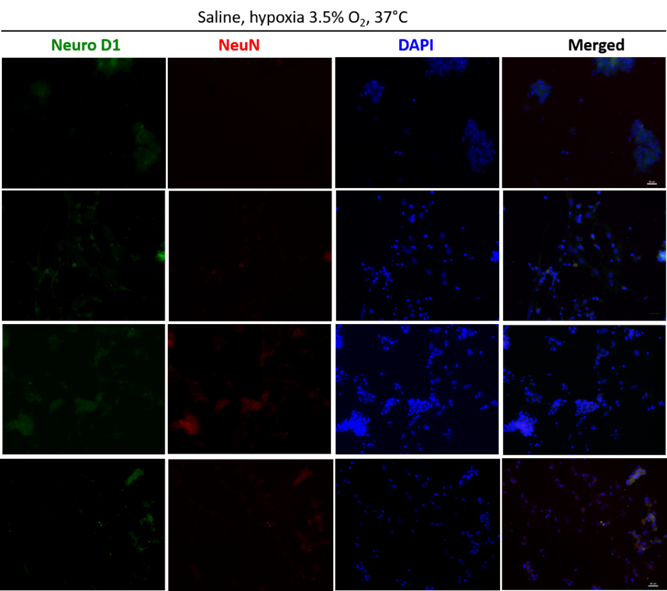


**Figure S3. Full blot images of representative blots of DRP1 and MFN2 used in figure 5.** Full blot images of DRP1 and B-Actin at 24 hrs and day 7 post MCAO (A), full blot images of MFN2 and B-Actin at 24 hrs and day 7 post MCAO (B).

**A**

**B**

**
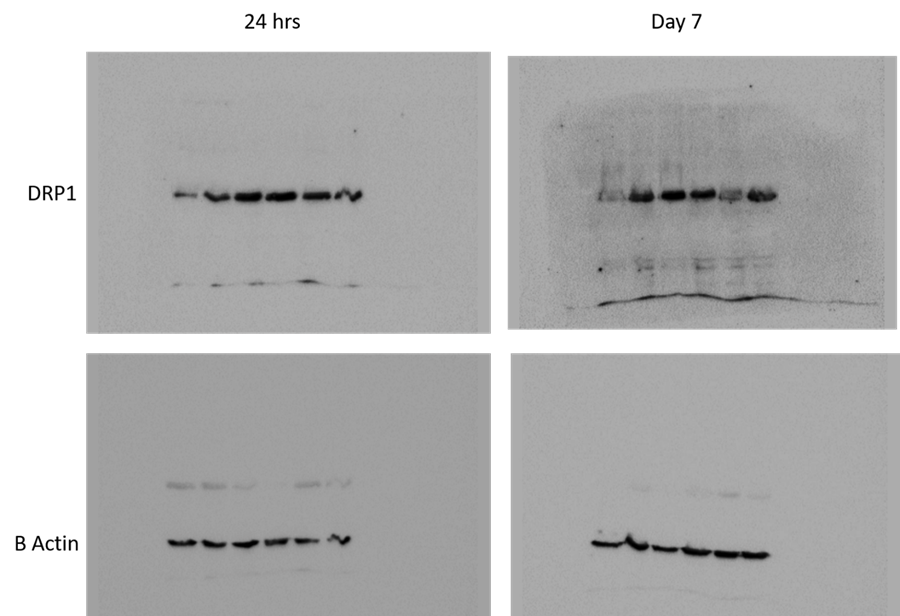
**


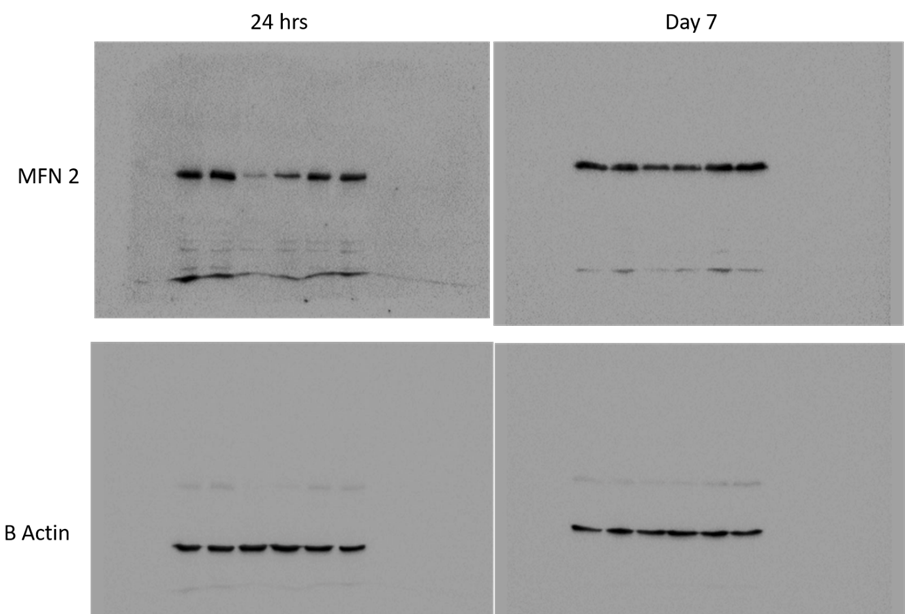


**Figure S4. Transmission electron micrographs of rat brain tissues of representative images used in figure 6.** Multiple electron microscopy images of randomly selected brain tissue sections of Sham (A), Vehicle (B) and Sovateltide (C) treated rats at 24 hrs and day 7 post MCAO.

1. **Sham**


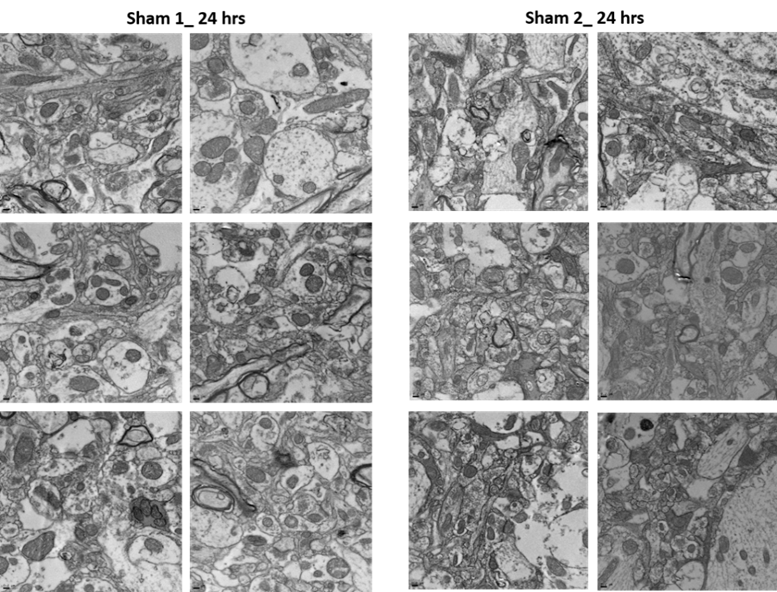


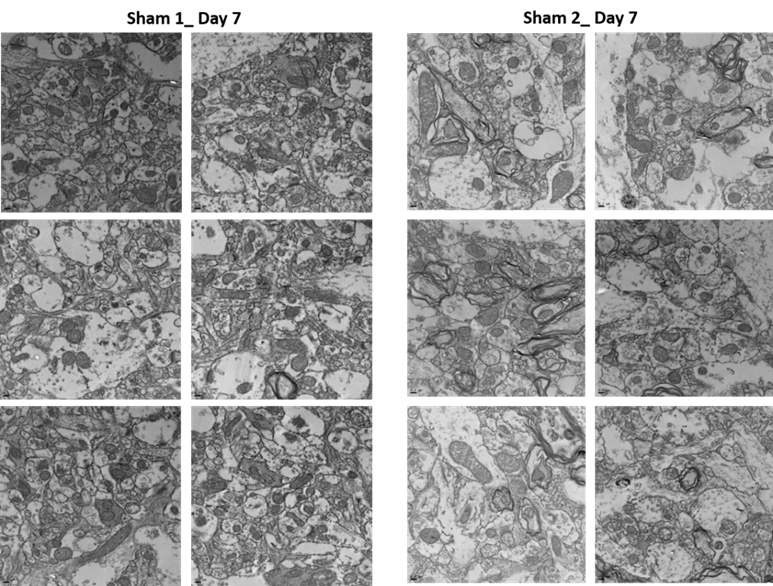


1. **Vehicle**


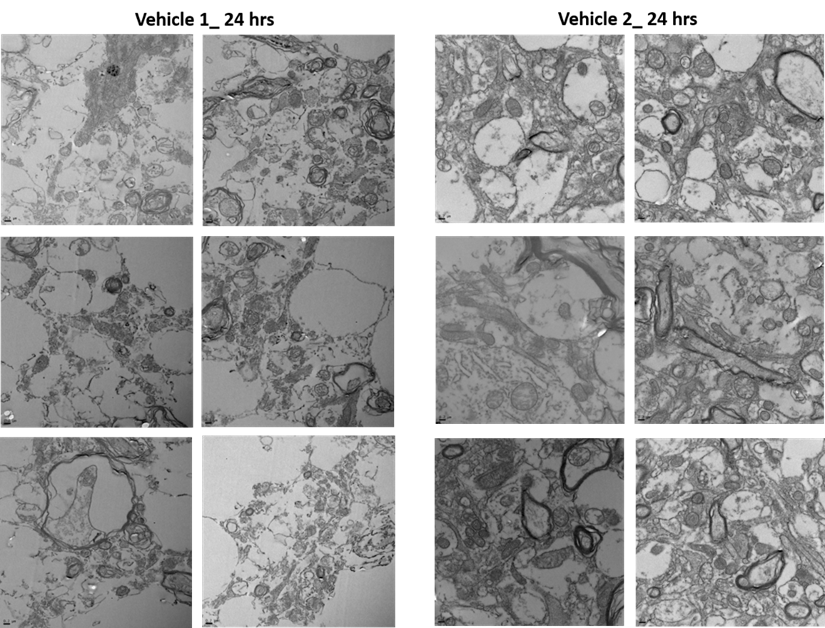


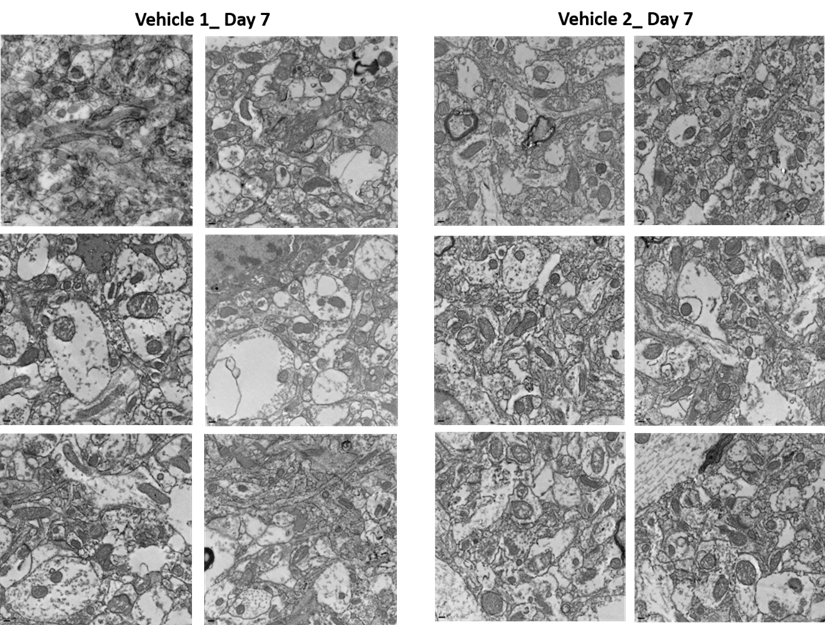


1. **Sovateltide**

**
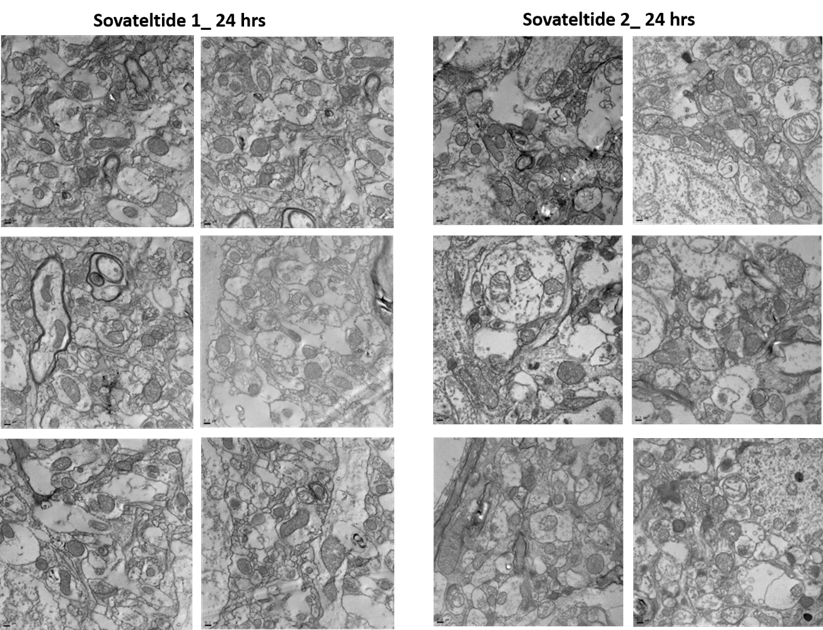
**


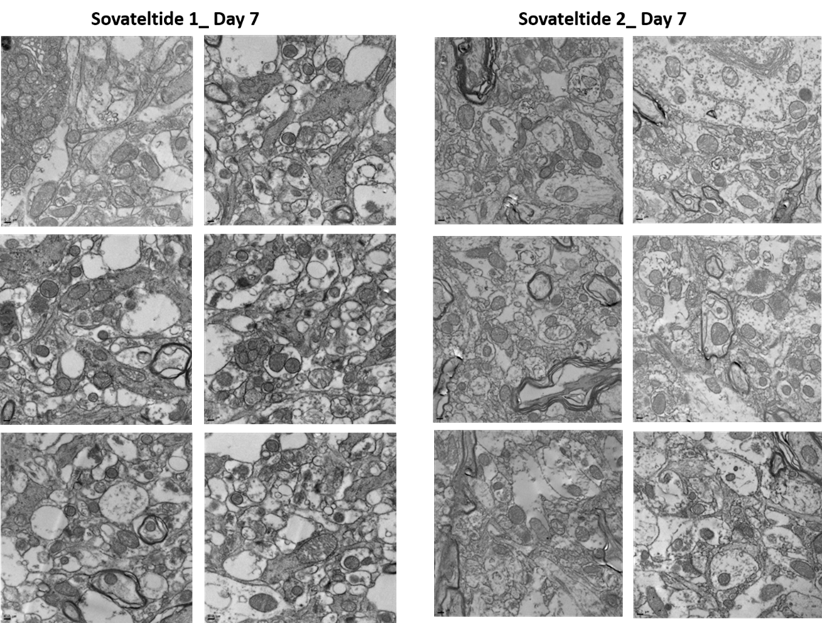


**Figure S5. Images of in situ PCR of MT-ATP8 DNA in rat brain tissues of representative images used in figure 7.** Microscopy images acquired from 3 randomly selected brain tissue sections of sham, vehicle and sovateltide treated 1^st^ set of rats (A), and 2^nd^ set of rats (B). MT-ATP8 (red).

1. Rat No. 1

Tissue area 1^st^ Tissue area 2^nd^ Tissue area 3rd


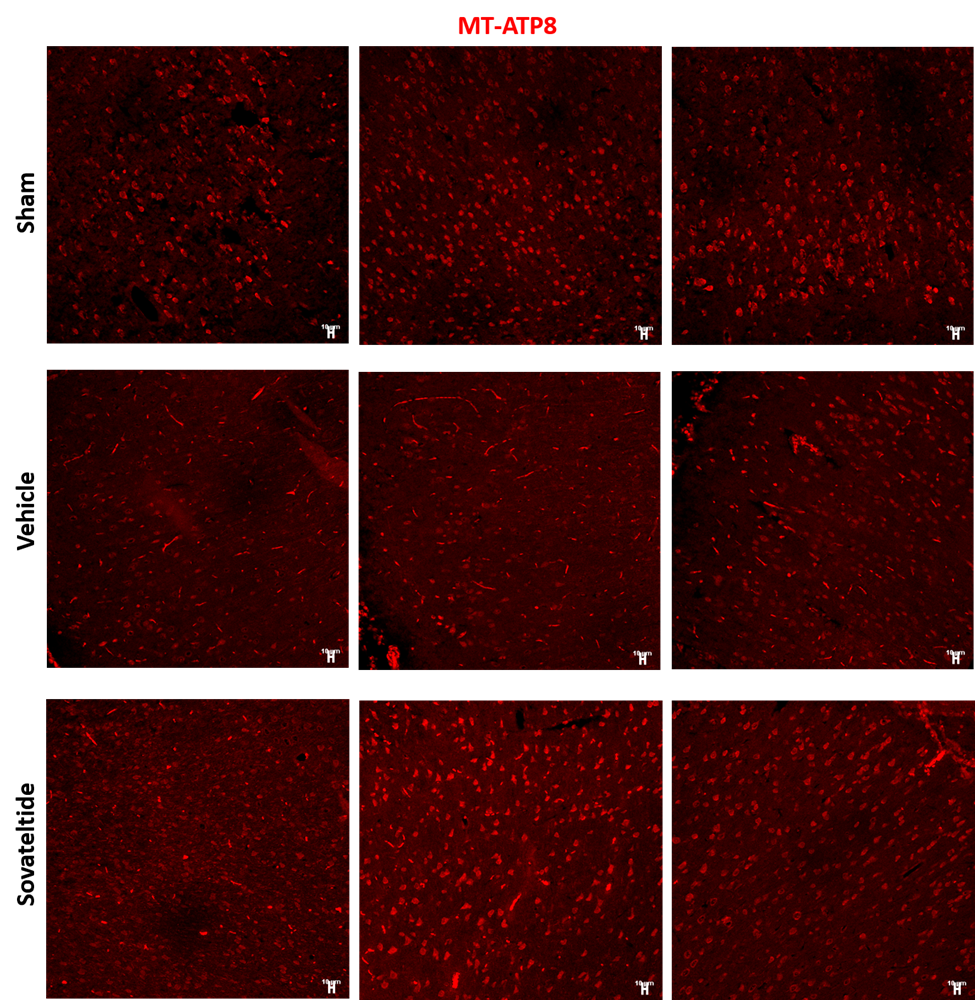


1. Rat No. 2

Tissue area 1^st^ Tissue area 2^nd^ Tissue area 3rd


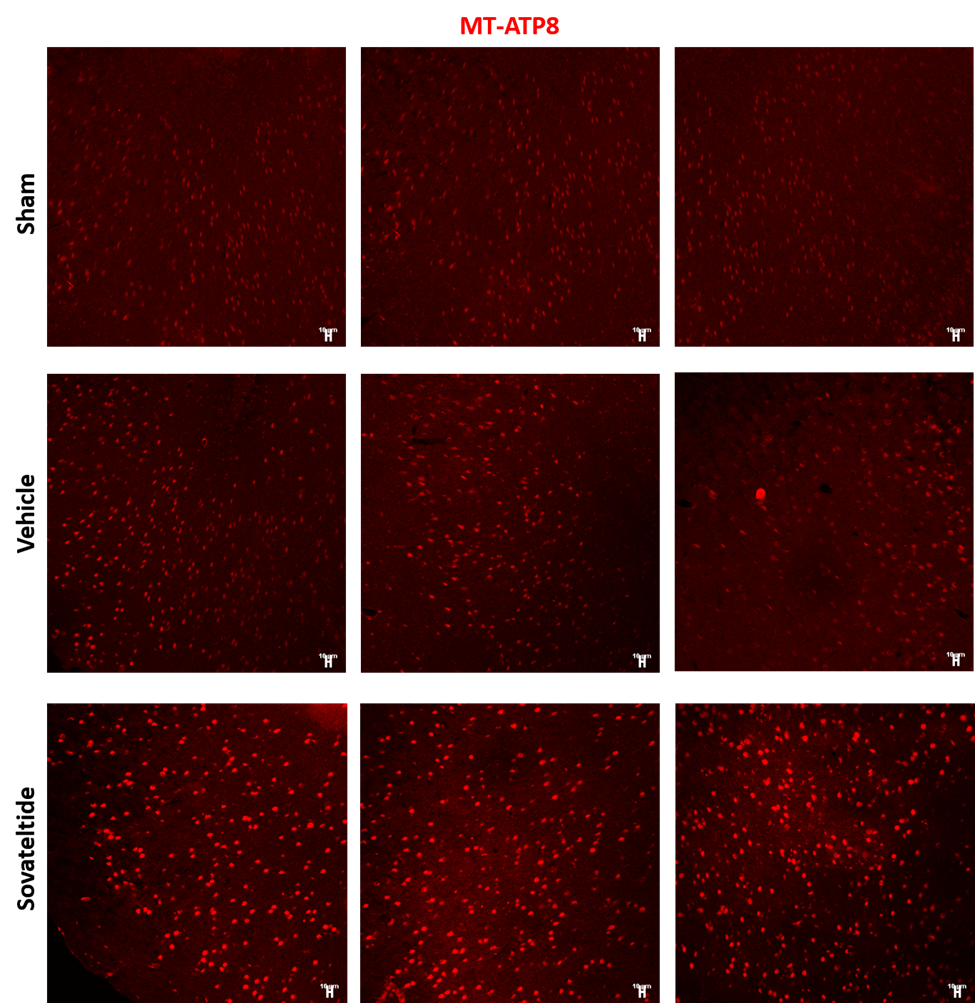

Supplement: Supplementary file 1 — Supplementary Information 1. [file 41598_2020_69673_MOESM1_ESM.docx]
